# Orexin Neurons to Sublaterodorsal Tegmental Nucleus Pathway Prevents Sleep Onset REM Sleep-Like Behavior by Relieving the REM Sleep Pressure

**DOI:** 10.34133/research.0355

**Published:** 2024-04-30

**Authors:** Hui Feng, Qi-Cheng Qiao, Qi-Fa Luo, Jun-Ying Zhou, Fei Lei, Yao Chen, Si-Yi Wen, Wen-Hao Chen, Yu-Jie Pang, Zhi-An Hu, Yi-Bin Jiang, Xu-Yang Zhang, Teng-Yuan Zhou, Xin-Yan Zhang, Nian Yang, Jun Zhang, Rong Hu

**Affiliations:** ^1^ Department of Neurobiology, Army Medical University, 400038 Chongqing, P.R. China.; ^2^ Department of Neurosurgery and Key Laboratory of Neurotrauma, Southwest Hospital, Army Medical University, 400038 Chongqing, P.R. China.; ^3^ Department of Physiology, Army Medical University, 400038 Chongqing, P.R. China.; ^4^ Sleep Medicine Center, West China Hospital, Sichuan University, 610000 Chengdu, Sichuan, P.R. China.

## Abstract

Proper timing of vigilance states serves fundamental brain functions. Although disturbance of sleep onset rapid eye movement (SOREM) sleep is frequently reported after orexin deficiency, their causal relationship still remains elusive. Here, we further study a specific subgroup of orexin neurons with convergent projection to the REM sleep promoting sublaterodorsal tegmental nucleus (OX^SLD^ neurons). Intriguingly, although OX^SLD^ and other projection-labeled orexin neurons exhibit similar activity dynamics during REM sleep, only the activation level of OX^SLD^ neurons exhibits a significant positive correlation with the post-inter-REM sleep interval duration, revealing an essential role for the orexin-sublaterodorsal tegmental nucleus (SLD) neural pathway in relieving REM sleep pressure. Monosynaptic tracing reveals that multiple inputs may help shape this REM sleep-related dynamics of OX^SLD^ neurons. Genetic ablation further shows that the homeostatic architecture of sleep/wakefulness cycles, especially avoidance of SOREM sleep-like transition, is dependent on this activity. A positive correlation between the SOREM sleep occurrence probability and depression states of narcoleptic patients further demonstrates the possible significance of the orexin-SLD pathway on REM sleep homeostasis.

## Introduction

The proper timing of vigilance states serves fundamental brain functions and is intricately orchestrated by brain-wide neuronal firing dynamics [1–4]. The hypothalamic orexin/hypocretin neurons are indispensable for sleep/wakefulness regulation [5]. It has been found that loss of these neurons leads to a fairly wide range of abnormalities in sleep/wakefulness behaviors [3,6]. Chief among them are deficits related to dysregulation of wakefulness state or rapid eye movement (REM) sleep, including excessive daytime sleepiness, frequent sleep–wakefulness transitions, cataplexy, vivid hallucinations, sleep paralysis, REM sleep behavior disorder (RBD), and sleep onset REM (SOREM) sleep [6–8]. Specifically, those REM sleep-related symptoms reflect that besides the direct deficiency in REM sleep consolidation, its homeostatic timing is also largely affected. Generally, the proper timing of a vigilance state is highly correlated with the homeostatic pressure for this state [9,10]. However, the exact relationship between the orexin neurons and the regulation of REM sleep pressure during sleep/wakefulness cycles remains largely elusive.

As a complicated regulatory system, different clusters of orexin neurons may utilize unique dynamics to orchestrate different processes of a single behavior [11,12]. In homeostatic regulation of sleep/wakefulness cycles, it has been accepted that activities of orexin neurons are subsequently relayed to many different neuronal circuitries that regulate distinct aspects of vigilance states [13,14]. Very prominently, the ahead firing increase and maximal activities of orexin neurons are thought to cope with arousal-related functions during wakefulness [15–18]. Researchers have indeed found that the orexin neurons’ active connection to the locus coeruleus (LC) is a critical integrator-effector circuit that regulates NREM sleep/wake behavior during the inactive period [19,20]. Intriguingly, the firing activities of orexin neurons and even orexin release have also been observed during REM sleep [11,17,18,20–22]. We have also found that the activity of orexin neurons can be relayed to the sublaterodorsal tegmental nucleus (SLD) to play an important role in REM sleep stabilization [22]. Therefore, whether the orexin neurons to SLD neural pathway is specifically related to REM sleep pressure during sleep/wakefulness cycles, as well as its potential functional significance, needs to be clarified.

In the present study, through a projection-based viral-genetic labeling method, we have first observed the projection architecture of orexin neurons that targeted the SLD (OX^SLD^ neurons) and identified that this orexin subpopulation preferentially projected with fewer axon collaterals. Using fiber photometry, the REM sleep-related neuronal dynamics of the OX^SLD^ and other projection-labeled orexin neurons were next observed during sleep/wakefulness cycles for correlating them with different physiological REM sleep parameters. A significant positive correlation was selectively found between the activation level of OX^SLD^ neurons and the post-inter-REM sleep interval durations, indicating an essential role in relieving REM sleep pressure for the orexin neurons to SLD neural pathway during sleep/wakefulness cycles. Through combining monosynaptic tracing, we further found that multiple inputs may help shape this specific REM sleep-related dynamics of OX^SLD^ neurons. Finally, genetic ablation of this pathway demonstrated abnormal REM sleep architectures in sleep/wakefulness cycles, which were caused by the deficiency of both pressure relief and episode stability. Furthermore, the possible significance of the orexin-SLD pathway’s homeostatic regulation on REM sleep is demonstrated by the link between the increased REM sleep pressure, indicated by frequent SOREM sleep, and the high score of the self-rating depression scale (SDS) index from narcoleptic patients. These results thus reveal the specific significance of the neural pathway from orexin neurons to SLD for relieving REM sleep pressure and preventing related clinical disorders.

## Results

### Collateral projection characteristics of OX^SLD^ neurons

**Fig. 1.**
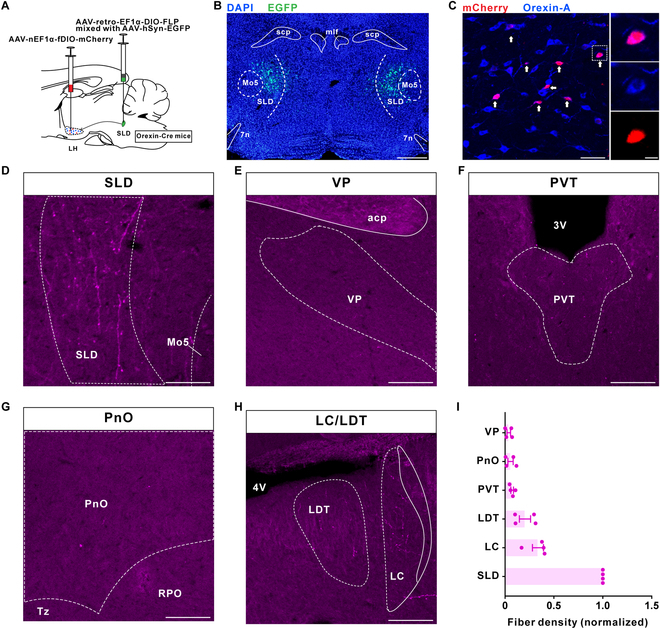
The projection of the orexin-SLD pathway is converged to mainly target the SLD. (A) Schematic drawing of virus injections. (B) A representative image showing the location of virus injections in the SLD. Scale bar, 500 μm. (C) Representative images showing the mCherry (red)-labeled OX^SLD^ neurons (purple) among the orexin neuronal entirety (blue). Scale bars, 50 μm (merged view) or 10 μm (magnified view). (D to H) Representative images of mCherry-labeled fibers in the SLD, VP, PVT, PnO, LC, and LDT. Scale bars, 100 μm. (I) Group data showing the relative fiber density of the OX^SLD^ neurons in different brain regions (*n* = 4 mice). Related to Fig. S3. scp, superior cerebellar peduncle; mlf, medial longitudinal fasciculus; 7n, facial nerve or its root; Mo5, motor trigeminal nucleus; acp, anterior commissure, posterior; 3V, third ventricle; Tz, nucleus of the trapezoid body; RPO, nucleus of the trapezoid body. Data are presented as mean ± SEM.

Projection-based viral-genetic labeling was first applied to label the OX^SLD^ neuronal population and a fluorescence marker was expressed in these neurons to examine their collateral projection patterns. This was achieved by bilateral injection of a retrograde-tracing virus AAV-retro-EF1α-DIO-FLP into the SLD of orexin-Cre mice, followed by bilateral injection of an anterograde-tracing virus AAV-nEF1α-fDIO-mCherry into the lateral hypothalamus (LH) (Fig. 1A and B and Fig. S1). We consistently observed OX^SLD^ neurons across the LH and varicose-like orexin nerve fibers arising from them throughout the SLD region (Fig. 1C and D and Fig. S2). Based on the reported four different pathways of orexin projections [13], in several important nodes (Fig. S3), the relative fiber density to the SLD of orexin projection was next calculated. Intriguingly, only very few collaterals were found in the ventral pallidum (VP) (3.8% ± 1.8%) of the ventral ascending pathway (Fig. 1E and I), pariventricular thalamus nucleus (PVT) (7.3% ± 1.3%) of the dorsal ascending pathway (Fig. 1F and I), and oral pontine reticular nucleus (PnO) (5.6% ± 2.7%) of the ventral descending pathway (Fig. 1G and I), indicating a concentrated fiber direction of OX^SLD^ neurons within the dorsal descending pathway. Moreover, even within this pathway, OX^SLD^ neuronal collaterals were much fewer detected in the LC (33.7% ± 5.6%) and the laterodorsal tegmental nucleus (LDT) (20.4% ± 5.8%) than orexin nerve fibers in the SLD (Fig. 1H and I). Together, the orexin-SLD neuronal pathway seems to be preferentially projecting to the SLD with fewer axon collateral, indicating a specific REM sleep relevance of the OX^SLD^ neurons.

### Functional activity dynamics of OX^SLD^ neurons during natural sleep/wakefulness cycles

**Fig. 2.**
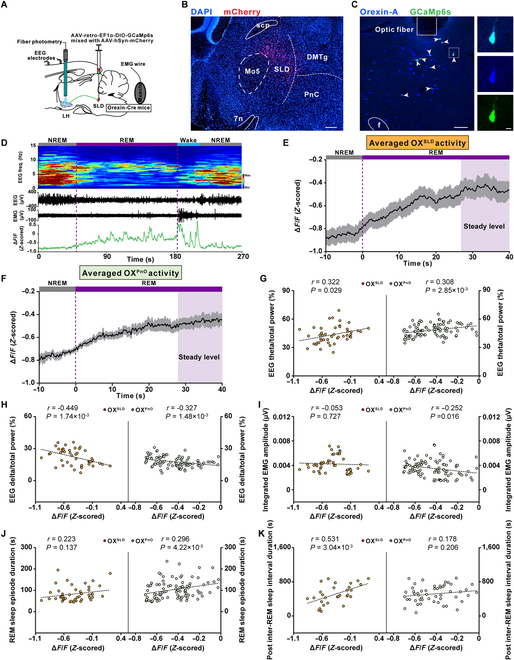
The averaged activity of OX^SLD^ neurons gradually increases to a steady level during REM sleep to specifically relieve REM sleep pressure. (A) Schematic drawing of fiber photometry recordings in the LH OX^SLD^ neurons. (B) A representative image showing virus injection sites in the SLD. Scale bar, 200 μm. (C) Representative images showing GCaMp6s^+^ expression (green) in orexin-A^+^ neurons (blue). Scale bars, 100 μm (merged view) or 10 μm (magnified view). (D) Representative color-coded hypnogram, EEG spectrogram, EEG/EMG traces, and GCaMP fluorescence trace of OX^SLD^ neurons recorded simultaneously in a REM sleep episode. (E) Averaged OX^SLD^ neuronal activity during the REM sleep episodes (*n* = 7 mice). Shadow represents SEM. (F) Averaged OX^PnO^ neuronal activity during the REM sleep episodes (*n* = 9 mice). Shadow represents SEM. (G to J) Left panels showing correlations between the steady activation level of OX^SLD^ neurons during REM sleep episodes and the corresponding EEG theta/total power (G), delta/total power (H), integrated EMG amplitude (I), and REM sleep episode duration (J) (*n* = 46 episodes from 7 mice). Right panels for OX^PnO^ neurons (*n* = 92 episodes from 9 mice). Dashed line, linear fit. (K) Left panel showing the correlation between the steady activation level of OX^SLD^ neurons during REM sleep episodes and the uniformly generated post-inter-REM sleep interval duration (*n* = 29 episodes from 7 mice). Right panel for OX^PnO^ neurons (*n* = 52 episodes from 9 mice). Dashed line, linear fit.

Fiber photometry was next employed to record sleep/wakefulness-related neuronal dynamics of this potential function-unique OX^SLD^ subgroup. A retrograde tracing virus AAV-retro-EF1α-DIO-GCaMp6s was injected into the SLD of orexin-Cre mice to selectively express GCaMp6s on the OX^SLD^ neurons, followed by electroencephalogram (EEG)/electromyogram (EMG) electrodes and optical fiber implantations in the LH (Fig. 2A to C and Fig. S4). We found that these neurons were active and exhibited large fluctuations across the entire sleep/wakefulness cycles (Fig. S5A). Specifically, OX^SLD^ neuronal activity time-lockly increased at sleep (including NREM and REM sleep) to wakefulness transitions and decreased at wakefulness to NREM sleep transitions (Fig. S5B to D), similar to the reported properties of orexin entirety [23]. In addition to these wakefulness-related activities, intriguingly, OX^SLD^ neurons were also activated at NREM to REM sleep transitions (Fig. S5E). Moreover, we found that OX^SLD^ neurons showed the lowest activity level at ~10 to 20 s before the NREM to REM sleep transitions, and the activity seemed to gradually increase and finally maintain at an active level throughout REM sleep (Fig. S5C and E). These results indicate that OX^SLD^ neurons indeed exhibit a REM sleep-related activity pattern, in addition to the common wakefulness-related activity for the orexin entirety.

We further focused on the detailed activity characteristics of OX^SLD^ neurons during REM sleep episodes. After transitioning into REM sleep, the basal activity of OX^SLD^ neurons exhibited a slow increasing trend accompanied by many randomly distributed GCaMp6s transients, and the baseline seemed to reach a relatively stable level if the episode was long enough (Fig. 2D). We thus averaged the activity of REM sleep episodes with duration longer than 40 s to more clearly visualize the activity pattern, and found that the averaged activity of OX^SLD^ neurons exhibited a long-lasting increasing pattern, which reached a relatively steady level at ~30 s after transition into REM sleep (Fig. 2E, see Materials and Methods). This highest-level activity stably lasted to the end of REM sleep, and would not further increase with prolonged REM sleep episodes (Fig. 2E and Fig. S6). To examine essential functions of the orexin-SLD pathway in REM sleep during sleep/wakefulness cycles, this steady activation level was next used to correlate with core features of REM sleep (see Materials and Methods). We found that there was a significant positive and negative correlation between this activation level and the EEG theta component (theta/total power) and delta component (delta/total power) of corresponding REM sleep episodes from the recorded 7 mice, respectively (Fig. 2G and H, left). No significant correlation appeared between this activation level and the integrated EMG amplitude (Fig. 2I, left). These results suggest that the activity of the orexin-SLD pathway may actively participate in regulating REM sleep and its functions during natural sleep/wakefulness cycles. We therefore analyzed the correlation degree between this steady activation level and the REM sleep performances during sleep/wakefulness cycles. The correlation between this REM sleep-related OX^SLD^ neuron activation level and the episode duration was not significant (Fig. 2J, left). Next, we explored its potential contribution to the relieving of REM sleep pressure. The pressure changes during REM sleep were reflected by a group of uniformly generated post-inter-REM sleep interval durations (see Materials and Methods). A significant positive correlation emerged between the OX^SLD^ neuron activation level and the post-inter-REM sleep interval duration (Fig. 2K, left). All these analyses thus demonstrate that the activity of the orexin-SLD pathway should substantially contribute to REM sleep homeostasis through a dominant action on relieving REM sleep pressure.

We next recorded activities from another orexin neuronal subgroup projecting to the PnO (OX^PnO^ neurons), which is also a REM sleep-promoting nucleus [24,25], to explore the distinct roles of different subgroup of orexin neurons in REM sleep. Compared with OX^SLD^ neurons, there were almost no differences in the activity dynamics during sleep/wakefulness transitions and REM sleep-related activity patterns recorded from OX^PnO^ neurons (Fig. 2F and Figs. S7 and S8). In OX^PnO^ neurons, a significant positive and negative correlation were also found between their stable activation level and the corresponding EEG theta component (theta/total power) and delta component (delta/total power), respectively (Fig. 2G and H, right). Moreover, this activation level was negatively correlated with the integrated EMG amplitude (Fig. 2I, right). Consequently, there was a significant positive correlation between this activation level and REM sleep episode duration (Fig. 2J, right). However, intriguingly, no significant correlation was detected between their steady activation level and the post-inter-REM sleep interval duration (Fig. 2K, right). Therefore, these results indicate that the activity of the orexin-SLD pathway may play a specific role in relieving REM sleep pressure during natural sleep/wakefulness cycles (Fig. S9).

### Monosynaptic inputs to OX^SLD^ neurons and their relation to functional OX^SLD^ neuronal activation during REM sleep

**Fig. 3.**
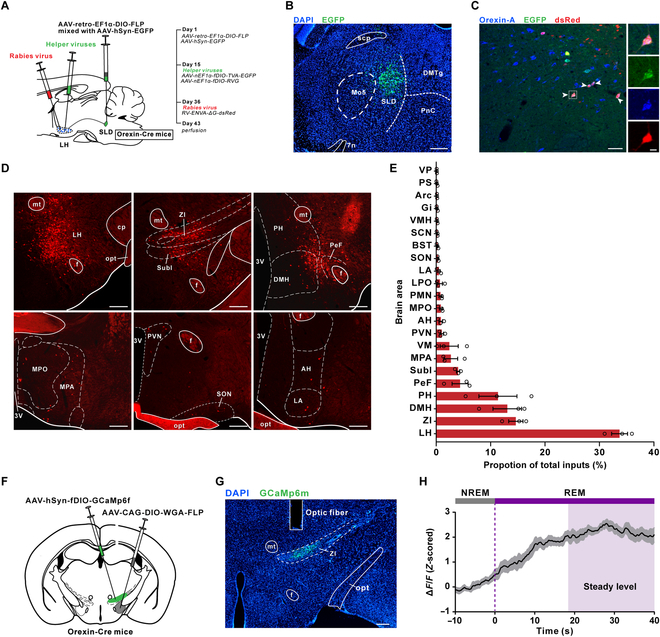
Multiple inputs modulate the REM sleep-related activation of the OX^SLD^ neurons. (A) Schematic of virus injections showing monosynaptic input tracing of OX^SLD^ neurons. (B) A representative image showing the location of virus injection in the SLD. Scale bar, 200 μm. (C) Representative images showing that the majority of identified OX^SLD^ neurons (orexin-A^+^ and EGFP^+^) are successfully transfected as starter cells (orexin-A^+^, dsRed^+^, and EGFP^+^). Scale bars, 50 μm (merged view) or 10 μm (magnified view). (D) Representative images showing several brain regions containing high level of RV-dsRed-labeled input neurons to the OX^SLD^ neurons. Scale bars, 200 μm. (E) Group data showing the proportion of monosynaptic inputs to OX^SLD^ neurons in different brain regions (*n* = 3 mice). (F) Schematic drawing showing fiber photometry recordings in the orexin-projecting ZI neurons. (G) A representative image showing the location of virus injection and optical fiber in the ZI. Scale bar, 200 μm. (H) Averaged ZI neuronal activity during the REM sleep episodes (*n* = 6 mice). Shadow represents SEM. f, fornix; mt, mammillothalamic tract; opt, optic tract; cp, cerebral peduncle; SubI, subincertal nucleus; DMH, dorsomedial hypothalamic nucleus; PH, posterior hypothalamic area; PeF, perifornical nucleus; MPO, medial preoptic nucleus; MPA, medial preoptic area; PVN, paraventricular hypothalamic nucleus; SON, supraoptic nucleus; LA, lateroanterior hypothalamic nucleus; AH, anterior hypothalamic area; VM, ventromedial thalamic nucleus; PMN, paramedian reticular nucleus; LPO, lateral preoptic area; BST, bed nucleus of the stria terminalis; SCN, suprachiasmatic nucleus; VMH, ventromedial hypothalamic nucleus; Gi, gigantocellular reticular nucleus; Arc, arcuate hypothalamic nucleus; PS, parastrial nucleus. Data are presented as mean ± SEM.

To investigate the potential input origins for the functional activity of OX^SLD^ neurons, we employed a modified rabies virus-based monosynaptic tracing strategy that allowed us to visualize input neurons of the OX^SLD^ neurons (Fig. 3A and B). After confirming the majority of OX^SLD^ neurons as starter cells (Fig. 3C), we found that a fairly wide range of brain areas contained neurons monosynaptically linking with OX^SLD^ neurons (Fig. 3D and E), a property similar to the reported orexin entirety [26]. However, the quantity constitution of input neurons to OX^SLD^ neurons or the orexin entirety was different. The majority (>10%) of input neurons to OX^SLD^ neurons were detected in the LH, zona incerta (ZI), dorsalmedial hypothalamus (DMH), and perifornical hypothalamus (PH) (Fig. 3E). After comparing these major inputs to OX^SLD^ neurons and those to the orexin entirety reported by a previous study [26], we noticed that ZI provided a pronounced higher level of monosynaptic inputs to the OX^SLD^ subgroup, but not the orexin entirety. This difference suggests that ZI might be a unique input for the functional activity of the OX^SLD^ subgroup. We thus retrogradely expressed a GCaMp6s protein in those orexin-projecting ZI neurons to examine whether their activities could contribute to the functional activity of OX^SLD^ neurons (Fig. 3F and G). We found that the averaged activity of these ZI neurons gradually increased and reached a steady level at around 20 s after transitioning into REM sleep, and this stable activity was maintained until the REM sleep terminated (Fig. 3H and Fig. S10A to C). Further correlation analysis also showed that this ZI activation level was correlated with neither REM sleep episode duration, nor the post-inter-REM sleep interval duration (Fig. S10D to H), and these orexin-projecting ZI neurons contained neurotransmitter GABA (Fig. S10I), but not MCH (Fig. S11). The REM sleep-related activity of these ZI neurons has different characteristics and roles compared to OX^SLD^ neurons, suggesting that the inputs from ZI could actively participate to influence the functional activity of OX^SLD^ neurons, and thus, multiple origins can together help shape the REM sleep-related functional dynamics of OX^SLD^ neurons.

### Behavioral consequences on the REM sleep after genetic ablation of OX^SLD^ neurons

**Fig. 4.**
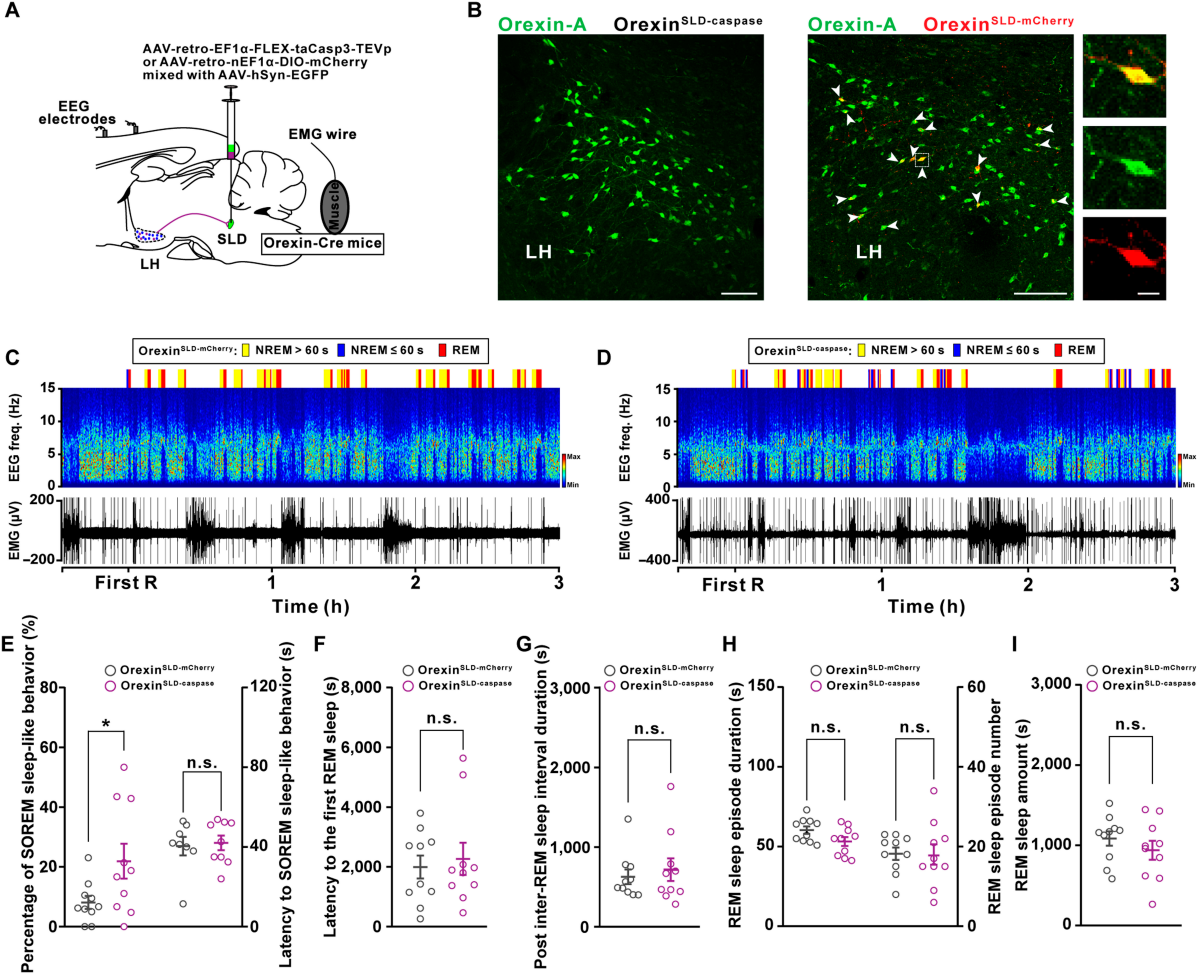
Genetic ablation of the orexin-SLD pathway increases the percentage of SOREM sleep-like behavior during sleep/wakefulness cycles. (A) Schematic drawing of virus injections in orexin-Cre mice showing genetic ablation of OX^SLD^ neurons. (B) Representative images showing orexin-A^+^ (green) neurons in the LH of orexin^SLD-caspase^ mice (left panel) and viral infections of mCherry^+^ (red) in the LH orexin-A^+^ (green) neurons of orexin^SLD-mCherry^ mice (right panel). Scale bars, 100 μm (merged view) or 10 μm (magnified view). (C and D) Representative color-coded hypnogram, EEG spectrograms, and EMG traces from an orexin^SLD-mCherry^ mouse (C) and an orexin^SLD-caspase^ mouse (D). (E to G) Changes of the percentage of SOREM sleep-like behavior and the latency to SOREM sleep-like behavior (E), the latency to the first REM sleep (F), and the post-inter-REM sleep interval duration (G) (*n* = 10 mice for each group). (H) Comparisons of the REM sleep episode duration (left) and number (right) (*n* = 10 mice for each group). (I) Comparison of the REM sleep amount (*n* = 10 mice for each group). Data are presented as mean ± SEM. *P < 0.05, two-tailed unpaired *t* test (E, left).

Functional significance of the orexin-SLD pathway in the REM sleep during sleep/wakefulness cycles was investigated after ablating OX^SLD^ neurons. A retrovirus carrying taCasp3 was bilaterally injected into the SLD of orexin-Cre mice (orexin^SLD-caspase^ mice) to selectively ablate OX^SLD^ subpopulation from the orexin entirety, followed by implantation of EEG/EMG electrodes (Fig. 4A and B). A group of orexin-Cre mice receiving bilateral AAV-retro-nEF1α-DIO-mCherry injections were set as control (orexin^SLD-mCherry^ mice). There were still abundant orexin neurons located in the LH after ablating OX^SLD^ neurons (Fig. 4B). Intriguingly, in this condition, the percentage of SOREM sleep-like behavior (defined by previous NREM sleep shorter than 60 s [27]) prominently increased from 8.1% ± 2.2% to 21.9% ± 5.8% during a 3-h recording period following the first REM sleep (Fig. 4C to E), and the mean latency from NREM sleep to SOREM sleep-like behavior was not affected (Fig. 4E, right), indicating insufficiently relieved pressure in sleep/wakefulness cycles, although no differences were detected in the latency to initiate the first REM sleep episode (Fig. 4F) or the averaged post-inter-REM sleep interval duration (Fig. 4G and Fig. S12). In addition, we found that REM sleep episode number or duration remained unchanged in this condition (Fig. 4H). Therefore, the total amount of REM sleep or other states in orexin^SLD-caspase^ mice all remained unaffected (Fig. 4I and Fig. S13). These data demonstrate the unique and indispensable role for the orexin-SLD pathway in maintaining REM sleep homeostasis, especially preventing the SOREM sleep-like behavior, through relieving REM sleep pressure during sleep/wakefulness cycles.

**Fig. 5.**
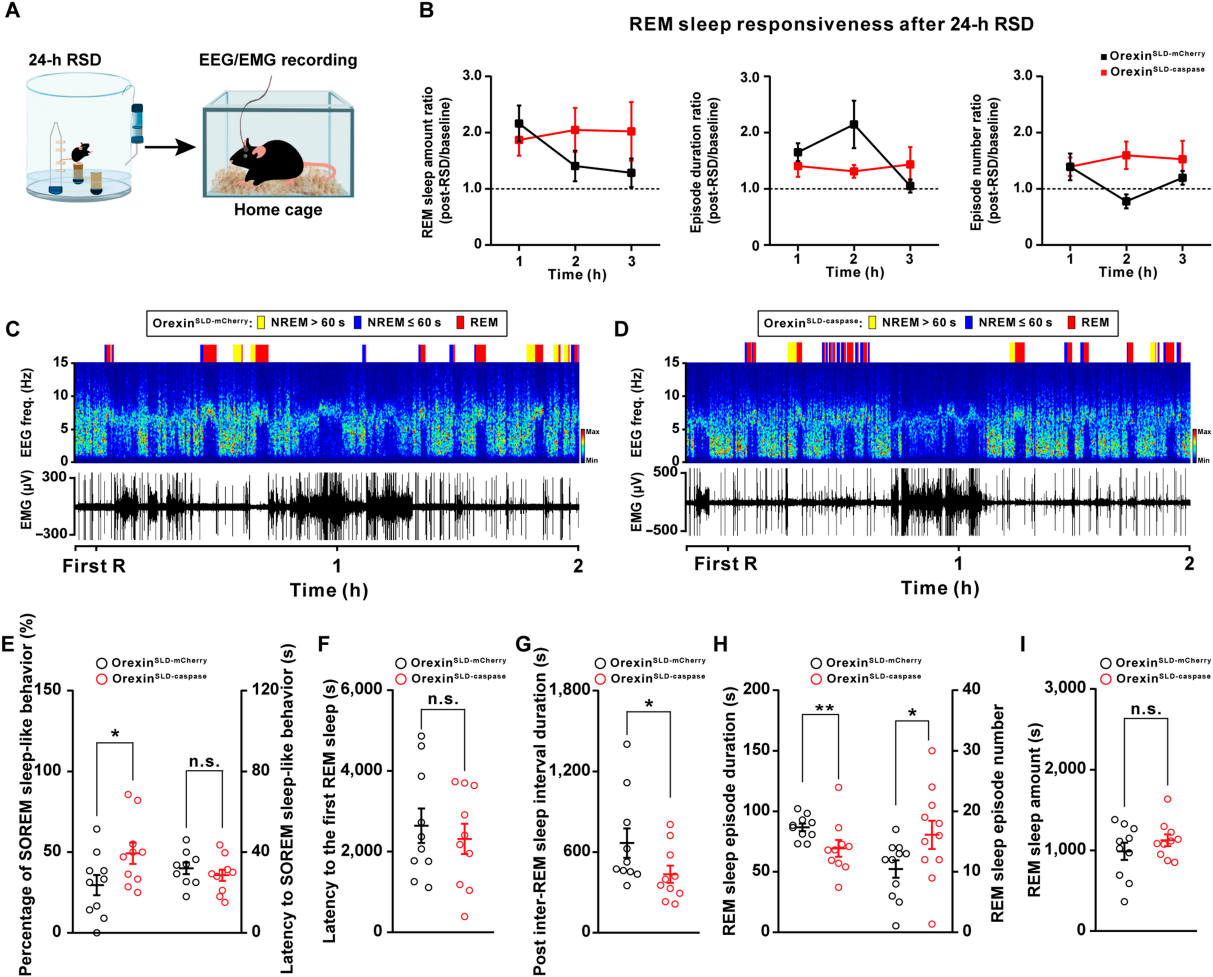
Loss of the orexin-SLD pathway leads to severe REM sleep fragmentation and a delayed rebound process when the physiological need for REM sleep elevates. (A) Schematic drawing of 24-h REM sleep deprivation (RSD) using the small-platforms-over-water method and EEG/EMG recordings in the home cage. (B) Trend graph of REM sleep responsiveness before and after 24-h RSD (*n* = 10 mice for each group). (C and D) Representative color-coded hypnogram, EEG spectrograms, and EMG traces during the rebound period after 24-h RSD from an orexin^SLD-mCherry^ mouse (C) and an orexin^SLD-caspase^ mouse (D). (E to G) Changes of the percentage of SOREM sleep-like behavior and the latency to SOREM sleep-like behavior (E), the latency to the first REM sleep (F), and the post-inter-REM sleep interval duration (G) during this rebound period after 24-h RSD (*n* = 10 mice for each group). (H) Comparisons of the REM sleep episode duration (left) and number (right) during this rebound period after 24-h RSD (*n* = 10 mice for each group). (I) Comparison of the REM sleep amount during this rebound period after 24-h RSD (*n* = 10 mice for each group). Data are presented as mean ± SEM. *P < 0.05, **P < 0.01, two-tailed unpaired *t* test (E and H, right) or two-tailed Mann–Whitney rank sum test (G and H, left).

We next evaluated whether the contribution of the orexin-SLD pathway in REM sleep homeostasis was more importantly required when REM sleep need was elevated, because this kind of elevation is a common feature crucial for several important brain functions, such as emotion regulation and energy balance [28,29]. To test this, we created a REM sleep high-need condition through REM sleep deprivation (RSD) for 24 h (Fig. 5A and Fig. S14; see Materials and Methods). Actually, this kind of high-need condition increased the percentage of SOREM sleep-like behavior from 9.9% ± 3.5% to 31.9% ± 7.0% in wild-type mice (Fig. S15). As shown in Tables S1 and S2 and Fig. 5B, the need to prolong REM sleep episodes was significantly higher in the first 2 h after deprivation in both orexin^SLD-mCherry^ and orexin^SLD-caspase^ mice, indicating a high REM sleep need during this 2-h period. Intriguingly, the steady activation level of the OX^SLD^ during REM sleep was also positively correlated with the post-inter-REM sleep interval duration during this period (Fig. S16). In addition, the method using to deprive REM sleep might induce some other interfering factors [30], but these possible interfering factors on the REM sleep may be comparable in both groups. Notably, after loss of this activation in orexin^SLD-caspase^ mice, the percentage of SOREM sleep-like behavior also significantly increased (Fig. 5C to E), and the mean latency from NREM to SOREM sleep was still not changed (Fig. 5E, right). There was still no difference in the latency to initiate the first REM sleep between the orexin^SLD-mCherry^ and orexin^SLD-caspase^ mice (Fig. 5F), but a significant decrease in the averaged post-inter-REM sleep interval duration emerged in this high-need condition (Fig. 5G). Further comparison showed that the decreased post-inter-REM sleep interval duration was mainly distributed in the duration ≤900 s (Fig. S17). In addition, a subsequently increased REM sleep episode number also appeared (Fig. 5H). We further observed a 20.0% decrease in the episode duration in the orexin^SLD-caspase^ mice even though REM sleep amount and other vigilance states were unchanged (Fig. 5H and I and Fig. S18), suggesting that the stabilization function of the orexin-SLD pathway also plays an important role in this REM sleep high-need condition. Together, REM sleep was severely fragmented during sleep/wakefulness cycles (Fig. 5C, D, and H) and the rebound process was largely delayed in this condition after ablating the orexin-SLD pathway (Tables S1 and S2 and Fig. 5B). Taken together, the contribution and necessity of the orexin-SLD pathway to REM sleep homeostasis further increased at high REM sleep need conditions.

### Comorbidity of narcolepsy in relation to SOREM sleep occurrence probability

**Fig. 6.**
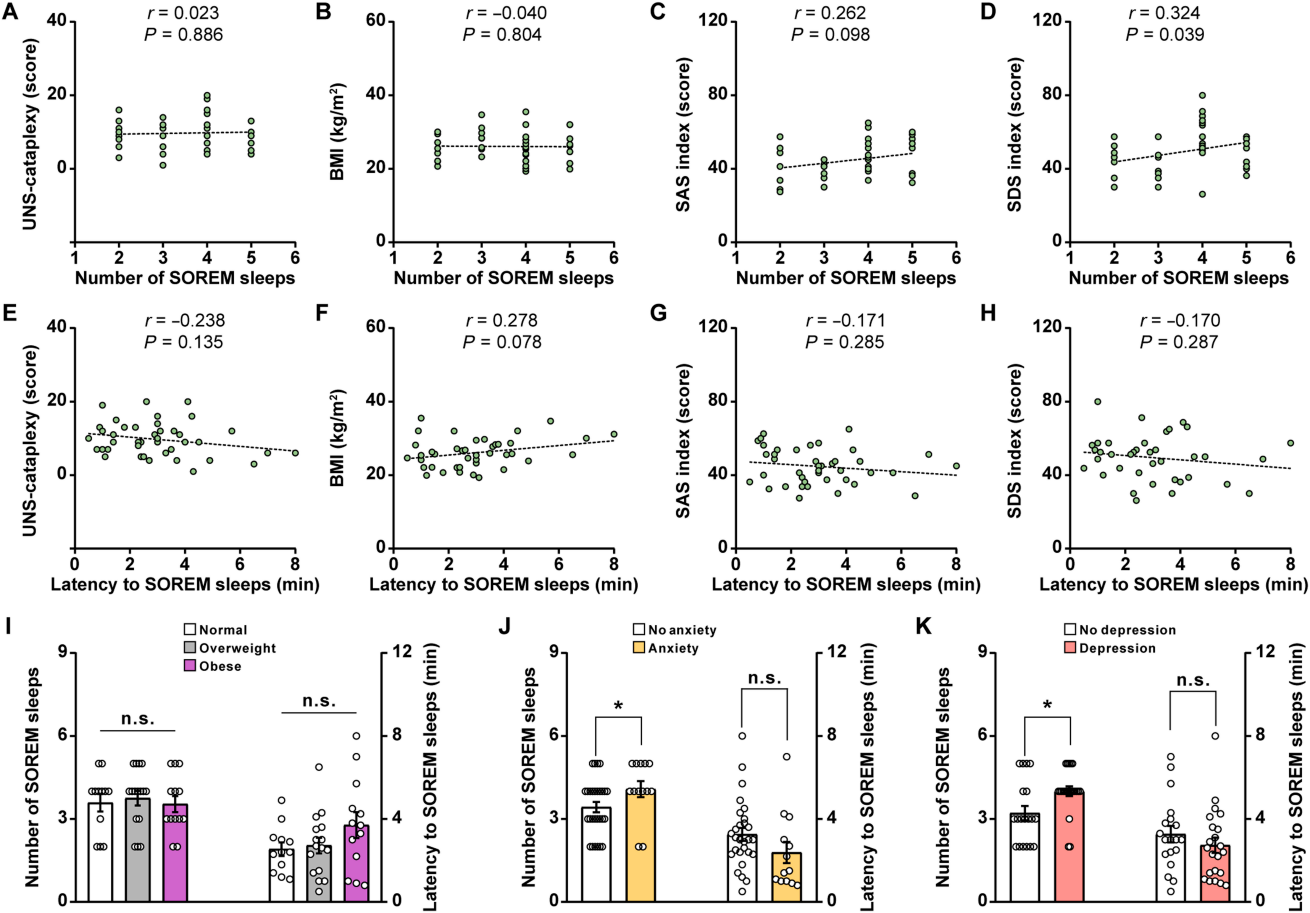
High SOREM sleep occurrence probability predicts a high risk for depression comorbidity in narcoleptic humans. (A to D) Correlation between the number of SOREM sleep and the corresponding UNS-cataplexy (A), BMI (B), SAS index (C), and SDS index (D). Each dot represents data from a patient (*n* = 41 patients). Dashed line, linear fit. (E to H) Correlation between the latency to SOREM sleep and the corresponding UNS-cataplexy (E), BMI (F), SAS index (G), and SDS index (H) (*n* = 41 patients). Dashed line, linear fit. (I to K) Changes of the number of SOREM sleep and the latency to SOREM sleep among different groups of patients [I left: *H* = 0.415, df = 2, *P* = 0.813; I right: *F*(2, 38) = 1.772, *P* = 0.099]. Data are presented as mean ± SEM. *P < 0.05, two-tailed Mann–Whitney rank sum test (J and K, left).

We further investigated the relationship between the SOREM sleep and risk factors for several comorbidities with high attack rate in narcoleptic patients, so as to investigate the important clinical significance of the disturbed REM sleep homeostasis after orexin-SLD pathway loss. Forty-one patients diagnosed with type I narcolepsy in the Sleep Medicine Center from West China Hospital were retrospectively reviewed. The demographics and clinical data were summarized in Table S3 (see Materials and Methods). In these narcoleptic patients, no correlation was detected between the number of SOREM sleep and the score of Ullanlinna Narcolepsy Scale (UNS)-cataplexy (Fig. 6A). Intriguingly, among all the screened comorbidity risk factors (Fig. 6B to D), a positive correlation between the number of SOREM sleep and the score of the SDS index was detected (Fig. 6D), demonstrating that the disturbed REM sleep pressure relief indicated by frequent SOREM sleep indeed contributed to the higher depression comorbidity risk in narcolepsy. Consistently, the narcoleptic patients indicated by the score of the SDS index for depression comorbidity (SDS index ≥ 50, *n* = 22/41) had a much higher number of SOREM sleep than patients without depression comorbidity (SDS index < 50, *n* = 19/41) (Fig. 6K, left), and this kind of difference in the number of SOREM sleep was also observed in narcoleptic patients with or without anxious comorbidity (Fig. 6J, left). The number of SOREM sleep among normal, overweight, and obese narcoleptic patients remained unchanged (Fig. 6I, left). These data suggest that the occurrence probability of SOREM sleep constitutes a specific and sensitive physiological marker for predicting depression risk in narcoleptic patients. In contrast, we noticed that there was no correlation when analyzing the latency from NREM to SOREM sleep with the score of UNS-cataplexy and all risk factors for screened comorbidities in narcoleptic patients (Fig. 6E to H). No differences can be detected in the latency from NREM to SOREM sleep for all comorbidities in narcoleptic patients (Fig. 6I to K, right).

## Discussion

The present study reveals an essential role for the orexin-SLD pathway in REM sleep homeostatic timing through actively relieving REM sleep pressure. Although the somas of OX^SLD^ neurons are diffusely distributed in the LH, their axons converge through a dorsal descending trajectory to target the SLD with few collaterals. In sleep/wakefulness cycles, OX^SLD^ neurons gradually increase their activities to a stably maintained activation state in REM sleep, which exhibits a significant positive correlation with the post-inter-REM sleep interval duration, and a significant correlation with REM sleep episode duration is not observed. This unveils a mechanism in active REM sleep pressure relief. Furthermore, selective ablation of this OX^SLD^ neuronal subpopulation consistently causes a unique REM sleep deficit characterized by insufficiently relieved pressure during daily REM sleep, and thus more SOREM sleep-like behavior. Importantly, when the REM sleep need is elevated, an increased influence of OX^SLD^ neuronal loss on daily REM sleep homeostasis is further demonstrated. Furthermore, the possible significance of the orexin-SLD pathway’s homeostatic regulation on REM sleep is demonstrated by the correlation between the increased REM sleep pressure and the higher risk for depression.

The observed morphological characteristics of the orexin-SLD pathway support its specific role in REM sleep regulation. Actually, this specificity is initially noticed by our previous report that a portion of orexin neurons are identified based on their projection to the SLD with a retrograde tracer and only a small fraction of them also innervates the neighboring LC [22]. Here, a further evaluation on this specificity is achieved by simultaneously labeling the OX^SLD^ neuron somas and mapping their collateral divergence after employing a double recombinase strategy in orexin-Cre mice. We consistently observe a diffused distribution pattern of OX^SLD^ neuron somas, unlike a proposed soma convergence in the lateral and medial LH, respectively, for different groups of projection-site defined orexin neurons [31–33]. However, intriguingly, axons arising from these diffused OX^SLD^ neuron somas are highly convergent. In fact, the convergence characteristic of orexin-containing axons has been noticed for the orexin entirety, which sends their axons mainly through four fiber bundles, and correspondingly, the orexin-SLD pathway goes through the dorsal descending one [13,34]. Consistently, we indeed observe few collaterals in all chosen important nuclei innervated by three other bundles (see Fig. 1E to G and I). This high convergent level of the orexin-SLD pathway is further supported by our observation that only a few collaterals are detected in the LC and LDT (see Fig. 1H and I), which are innervated by the same dorsal descending bundle [13]. All these findings thus support the notion that functionally distinct orexin neuron subpopulation can be classified based on their collateral projection patterns [31,32,35,36]. In contrast to the convergent projection pattern, monosynaptic inputs of OX^SLD^ neurons are found to arise from a fairly wide range of brain regions, similar to those of the orexin entirety [26,37]. Therefore, OX^SLD^ neurons may widely collect brain-wide input information like the orexin entirety [26,37], but the generated neuronal dynamics on them may interact with the SLD selectively to finally achieve specific roles during REM sleep.

The observed neuronal activity in OX^SLD^ neurons during REM sleep further indicates essential actions of the convergent orexin-SLD pathway in REM sleep regulation. Here, the OX^SLD^ neuronal subgroup arises the GCaMP6s signals at around entering REM sleep, and then the signals reach a stably maintained activation manner that lasts until REM sleep ends (see Fig. 2E). These results suggest that a REM sleep-related firing increase in individual OX^SLD^ neurons. Actually, this kind of firing increase during REM sleep is not prevalent for all orexin neurons. In contrast, orexin neurons projecting to the LH or basal forebrain (BF) may show further decreased firings in REM sleep as indicated by a study using fluorescent orexin-receptor probes in mice [15], although a micro-dialysis investigation finds increased orexin release during REM sleep in these regions in cats [20]. Actually, direct firing recordings of single orexin neuron show an occasional or intermediate firing pattern and even no firings during REM sleep [17,18,21], and a recent microendoscope observation reports that 32.1% of orexin neurons are REM sleep active [11], supporting all the related phenomenon including the activation or inhibition in a particular orexin subgroup during REM sleep. The neuronal substrates for these firing discrepancies are not clear. However, the intrinsic properties of orexin neurons, such as the KCNQ2/3 channel-mediated M current [23], would indeed change across different situations and may thus behave differently in different subgroups to affect their firings in different brain states. Furthermore, specifically to the OX^SLD^ neurons, diverse inputs with variable abundance and different activation kinetics in REM sleep, such as the ZI investigated in the present study [38], may also help shape the specific activation dynamics. Consequently, the sustained activation of these OX^SLD^ neurons finally emerges and should have its own specific role in REM sleep regulations.

Intriguingly, the sustained activation level from the OX^SLD^ neuronal subgroup is indeed positively correlated with EEG theta consolidation during REM sleep, demonstrating a direct effect of the orexin-SLD pathway on REM sleep features. This effect is in consistent with the reported roles of the SLD output in EEG and EMG during REM sleep [39,40] and the orexin-elicited synchronized excitation effect on the output of this pathway [22], although a significant correlation with EMG does not appear. Importantly, all these effects should provide necessary neuronal links for the orexin-SLD pathway to finally engage into daily REM sleep regulation [40,41]. Subsequently, observations from both normal and high REM sleep need sleep/wakefulness cycles all consistently confirm that this activation level is prominently and positively correlated with the post-inter-REM sleep interval duration (see Fig. 2K, left, and Fig. S13I), and meanwhile, no significant correlation is observed between this activation level and the REM sleep episode duration (see Fig. 2K, left, and Fig. S13I). Previously, REM sleep pressure is thought to be naturally dissipated during the episode, and only a positive but weak correlation between the REM sleep episode duration and the post-inter-REM sleep interval duration is employed to account for this neuronal process [42,43]. Our finding here, thus, for the first time, unveils that the relieving of REM sleep pressure is actively driven by specific brain mechanisms, including the activation of the orexin-SLD pathway. For REM sleep pressure regulation, only some mechanisms responsible for its accumulation have been noticed, such as increased level of brain-derived neurotrophic factor (BDNF) in the subcoeruleus region and the activation of MCH neurons in the LH [28,42,44,45]. Another aspect is that OX^PnO^ neurons are also found to be active during REM sleep and exhibit similar activation pattern with that of the OX^SLD^ neurons. However, in contrast, the activation level in OX^PnO^ neurons is only positively correlated with the REM sleep episode duration, and no significant correlation is found with the post-inter-REM sleep interval duration in sleep/wakefulness cycles (see Fig. 2J and K, right). It thus reflects that a certain degree of functional preference or even specialization may exist in different neuronal pathways arising from orexin neurons, even for the regulation of the same function. Intriguingly, a specific enhanced synchronized SLD output is elicited by orexin through directly exciting orexin receptor-positive neurons and increasing gap junction conductance among SLD neurons, which are electrically coupled [22]. This orexin-elicited synchronization largely helps recruit extensive coherence activities in multiple downstream REM sleep machineries of the SLD and thus further increases their coordination efficiency on an already active state for facilitation of REM sleep functions [39,40,46], which should be an important underlying mechanism for the role of orexin-SLD pathway in actively relieving REM sleep pressure.

The preferential role of the orexin-SLD pathway in relieving REM sleep pressure further indicates specific functional significance of the orexin-SLD pathway on daily REM sleep homeostasis. The actions of the orexin-SLD pathway should allow a reduced level of daily REM sleep pressure, which subsequently affects REM sleep homeostatic timing and architectures [43,47,48]. However, surprisingly, long-term loss of this pathway does not affect the first REM sleep latency and mean post-inter-REM sleep interval duration during sleep/wakefulness cycles. Meanwhile, the general REM sleep performances are also not affected in this condition. The unchanged performances seem different from effects generated by a 4-h chemogenetic inhibition of this pathway, which elicits a shortened REM sleep episode and a ~17% reduction in REM sleep amount [22]. It is likely that some compensations are elicited after the long-lasting loss of this neural pathway. Careful observations finally demonstrate that the ratio of SOREM sleep-like behavior is significantly increased in this condition (see Fig. 4E, left). This directly reveals the specific behavioral phenotype caused by insufficient pressure relieving due to loss of the orexin-SLD pathway in daily sleep cycles, demonstrating an irreplaceable role for this pathway in daily REM sleep homeostasis. Actually, the dysregulated REM sleep homeostatic timing including the SOREM sleep is indeed widely reported in narcolepsy [7,49,50]. It should also be noted that only SOREM sleep-like behavior, but not other kinds of disrupted timing such as cataplexy, occurs in the orexin^SLD-caspase^ mice, even at the high REM sleep need condition, indicating that additional influences on the occurrence of cataplexy from other players may exist, such as the lack of orexin’s direct modulation on central motor excitation [36,51–53]. Observations from our high REM sleep need model help reveal more important characteristics of the orexin-SLD pathway for REM sleep homeostasis. Unlike the moderate increase in SOREM sleep-like behavior during normal sleep, we observed that nearly half of REM sleep is SOREM sleep-like behavior with even shortened post-inter-REM sleep interval duration (see Fig. 5E, left, and G), which may finally cause increased REM sleep episode number (see Fig. 5H, right) in this challenged condition [47]. In addition, the stabilization role of the orexin-SLD pathway in this condition cannot be fully compensated, manifested as shortened REM sleep episode duration (see Fig. 5H, left) [22]. Due to both deficits in REM sleep timing and consolidation, severe REM sleep fragmentation and delayed response to REM sleep need finally appear in the orexin^SLD-caspase^ mice, implying the important significance of the orexin-SLD pathway in REM sleep homeostatic response. It should be noted that efficient homeostatic regulation of REM sleep is a crucial process for the homeostasis of several important functions, including emotion processing, motor control, and also energy balance [28,29]. Consistently, the disturbed REM sleep homeostasis has been reported to cause several related clinical disorders, such as depression, attention-deficit hyperactivity disorder (ADHD), and overweight [54–57]. Therefore, loss of the substantial contribution of the orexin-SLD pathway to REM sleep homeostasis may also account for a higher comorbidity rate of these disorders in narcolepsy.

More importantly, the exact clinical significance for the orexin-SLD pathway’s specific contribution in relieving daily REM sleep pressure is directly observed in the present study. Taking the opportunity to assess clinical data from type I narcoleptic patients with definite orexin deficiencies, a selective positive correlation indeed emerged between the occurrence probability of SOREM sleep and the degree of negative emotional states (see Fig. 6D), but not the metabolic levels measured by body mass index (BMI). It should be noted that frequent SOREM sleep has long been noticed to be associated with depressive symptoms in a variety of neurological disorders [58,59], and even major depression [60,61]. It seems that there exists a specific neuronal link between the insufficiently relieved REM sleep pressure, as indicated by frequent SOREM sleep, and the abnormal emotional regulations. Consistently, a previous report has directly observed that more intense emotional experience processing, especially the processing of negative emotional type, occurs during SOREM sleep compared with the normal ones [62]. Therefore, as a specific modulator of REM sleep pressure and thus the SOREM sleep, the vulnerability of the orexin-SLD neuronal pathway may constitute an important pathophysiological substrate for negative emotional dysregulations. Intriguingly, in those neurological disorders with high incidence for depression comorbidities, the deficiency of the orexin system or the SLD has been indeed reported [63–65]. Therefore, the occurrence probability of SOREM sleep sensitive to elevated REM sleep pressure constitutes a specific biomarker to predict risk of depression. The causal relationship of the orexin-SLD neuronal pathway with depression comorbidities needs to be further elucidated in the future. On the other hand, previous studies have tried to use the latency as a REM sleep parameter to predict depression occurrence and have found that this parameter is not reliable [66–68]. Consistently, our data have revealed that this latency does not significantly change when compared between the orexin^SLD-caspase^ and control mice, suggesting that this parameter of SOREM sleep would not sensitively change with the REM sleep pressure level, compared with the occurrence probability of SOREM sleep.

In summary, an active mechanism for relieving REM sleep pressure to prevent the occurrence of SOREM sleep-like behavior is revealed in the present study. The sensitivity of SOREM sleep in relation to depression and the specific modulatory role of the orexin-SLD pathway in SOREM sleep-like behavior provide insights for future clinical diagnosis and treatment. In addition, combining our previous report that demonstrates a REM sleep stabilization effect, this pathway’s multidimensional and coordinated functions in REM sleep regulation emerge. This working frame for orexin signaling to efficiently participate in a certain related behavior regulation through coordinating its different functional aspects fulfills its homeostatic regulation [35,69]. Moreover, the concept of a fine functional division in different orexin subgroups (see Fig. S9), as suggested here by the OX^SLD^ subgroup and wakefulness or reward-seeking behaviors by the OX^LC^ or OX^VTA^ subgroup [31], should also be further expanded and investigated in the orexin system.

## Materials and Methods

### Animals

Orexin-Cre mice (a gift from Luis de Lecea, Stanford University, USA) aged between 8 and 12 weeks were employed for all experiments. Mice were housed under a standard 12-h light/dark cycle (lights on at 8:00 AM, ZT 0; lights off at 8:00 PM, ZT 12) with free access to food and water. The environmental temperature was constantly kept at 22 ± 1 °C and the relative humidity was maintained at 50% ± 10%. All experimental procedures and animal care were executed according to the Guide for the Care and Use of Laboratory Animals of the Army Military Medical University.

### Virus injection

Mice were anesthetized with isoflurane (2% induction, 0.5% to 1.5% maintenance) and then fixed on stereotaxic platforms (RWD Life Science, China). Erythromycin eye cream was used to protect eyes from dryness and light damage. The midline scalp was incised to expose the skull. The following viruses were injected using a glass micro-pipette (tip diameter: ~20 μm) connected to the Nanoject II (Drummond Scientific, USA). Multiple 4.6-nl injections were made every 10 s and the glass pipette was left for 5 min to allow for diffusion and then withdrawn slowly. To specifically visualize the axon projection patterns of OX^SLD^ neurons, a mixture (1:1, 23 nl) of AAV-retro-EF1α-DIO-FLP and AAV-hSyn-EGFP (BrainVTA, China) was bilaterally injected into the SLD of orexin-Cre mice (bregma, AP: −5.10 mm, ML: ±0.95 mm, DV: −4.25 mm), followed by bilateral injection of an anterograde-tracing virus AAV-nEF1α-fDIO-mCherry (150 nl, BrainVTA, China) into the LH (bregma, AP: −1.55 mm, ML: ±1.05 mm, DV: −5.25 mm) after 2 weeks. AAV-hSyn-EGFP was used to indicate the infected region. To selectively record the activity of OX^SLD^ neurons, a mixture (1:1, 23 nl) of retrogradely transported AAV-retro-EF1α-DIO-GCaMp6s and AAV-hSyn-mCherry (BrainVTA, China) was injected into the SLD of orexin-Cre mice, and the AAV-hSyn-mCherry was used to indicate the injection sites. To map the monosynaptic inputs of OX^SLD^ neurons, a mixture (1:1, 23 nl) of AAV-retro-EF1α-DIO-FLP and AAV-hSyn-EGFP (BrainVTA, China) was injected into the SLD of orexin-Cre mice, and the AAV-hSyn-EGFP was used to indicate the infected region. After 2 weeks, a mixture (1:1, 150 nl) of AAV-helper (AAV-nEF1α-fDIO-TVA-EGFP and AAV-nEF1α-fDIO-RVG, BrainVTA, China) was injected into the LH of the same animal. Three weeks later, RV-ENVA-ΔG-dsRed (150 nl, BrainVTA, China) was injected into the LH of the same animal. To record the activity of orexin-projecting ZI neurons, AAV-CAG-DIO-WGA-FLP (150 nl, BrainVTA, China) was injected into the LH, followed by injection of AAV-hSyn-fDIO-GCaMp6f (18 nl, BrainVTA, China) in the ZI (bregma, AP: −1.40 mm, ML: ±0.95 mm, DV: −4.50 mm) of orexin-Cre mice. To genetically ablate OX^SLD^ neurons, a mixture (1:1, 23 nl) of AAV-retro-EF1α-FLEX-taCasp3-TEVp (OBiO, China) and AAV-hSyn-EGFP (BrainVTA, China) was injected into the SLD bilaterally. Mice injected with a mixture (1:1, 23 nl) of AAV-retro-nEF1α-DIO-mCherry and AAV-hSyn-EGFP (BrainVTA, China) were set as control. After injections, mice were recovered from anesthesia and housed in their home cages. Mice used for tracing the axon projection patterns or the monosynaptic inputs of OX^SLD^ neurons were sacrificed 1 to 2 weeks after virus injections.

### Surgery

For fiber photometry recording experiments, ceramic optical fibers (diameter: 200 μm, NA: 0.37, Inper, China) were implanted in the LH/ZI after 3 weeks of virus injections. For polysomnographic recordings, 2 EEG electrodes were implanted on the frontal (bregma, AP = +1.50 mm; ML = ±1.50 mm) and entorhinal cortex region (bregma, AP = −3.0 mm; ML = ± 2.0 mm). The 2 EMG electrodes were inserted into the neck muscles. All electrodes were permanently soldered to a connector that was then fixed to the skull with dental cement.

### Immunohistochemistry

Mice were anesthetized by isoflurane followed by sodium pentobarbital (150 mg/kg, i.p.). Then, mice were perfused transcardially with saline, followed by 4% paraformaldehyde. After removal, brains were post-fixed in 4% paraformaldehyde for 4 to 6 h and then placed in 30% sucrose in phosphate buffer solution (PBS) at 4 °C until they sank completely. Brains were sectioned coronally at 30 μm using a freezing microtome (CM 3050S, Leica). The slices containing interested brain regions were sequentially incubated with the primary and secondary antibodies according to these instructions. The following primary antibodies were used in this study: rabbit anti-orexin-A (1:2,000, AB3704, Millipore, USA) and rat anti-mCherry (1:1,000, M11217, Thermo Fisher, USA). The secondary antibodies used in the present study were Alexa Fluor 488 donkey anti-rabbit IgG (1:500, A21206, Thermo Fisher, USA), Alexa Fluor 405 donkey anti-rabbit IgG (1:500, AB175651, Abcam, USA), and Alexa Fluor 647 donkey anti-rat IgG (1:500, AB150155, Abcam, USA). Slices were mounted onto the microscope slides and coverslipped with the Fluoroshield-containing DAPI mounting media (F6057, Sigma-Aldrich, USA) or the Fluormount G mounting media (0100-01, SouthernBiotech, USA). Histological processes were also used to evaluate the virus-infected region and the sites of optical fiber implantations. If the locations were not correct, the related data would be excluded.

### Image acquisition and quantification

The immunofluorescent images were acquired with an LSM 900 (Carl Zeiss, Germany) or a virtual microscopy slide scanning system (VS200, Olympus, Japan). Then, they were further analyzed by the OlyVIA software (Olympus, Japan) or the Zen software 2012 (Carl Zeiss, Germany). For quantification of the axon fiber density of OX^SLD^ neurons or orexin neurons in the SLD, VP, PVT, PnO, LC, and LDT, images were collected with identical imaging parameters. The fluorescence values in these nuclei were then measured by Image Pro Plus (Media Cybernetics, USA). Briefly, coronal images containing these nuclei were first aligned to the mouse brain atlas [70]. The fluorescent axon terminals were next automatically identified based on their difference to the background. Then, the density of axon terminals in each nucleus was calculated as the ratio of the area occupied by OX^SLD^ neuronal fibers or orexin-immunoreactive fibers to the total area of the corresponding brain regions. The relative fiber density to the SLD of orexin projection was finally obtained. For tracing the monosynaptic inputs of OX^SLD^ neurons, RV-dsRed labeled neurons were counted in different brain regions according to the mouse brain atlas [70]. The profile of monosynaptic input was presented as the number of RV-dsRed-labeled neurons in a specific brain region to the total number of RV-dsRed-labeled neurons in the whole brain.

### EEG/EMG recordings and analyses

After the surgery, mice were allowed to recover in their home cages for 5 to 7 days and connected to the EEG/EMG connection cables and an optical fiber patch cord for adaption at least 2 days. On the recording day, the EEG/EMG recordings started at ZT 4 (12:00 AM) and ended at ZT 8 (4:00 PM). The EEG/EMG signals were recorded with a sampling rate of 1,000 Hz using the omniplex neural data acquisition system (PlexControl 1.10, Plexon, USA). These collected signals were next band-pass filtered (EEG: 1 to 20 Hz, EMG: 20 to 100 Hz).

Based on spectral signatures of EEG/EMG waveforms in 5-s epochs, EEG/EMG signals were classified into different vigilance states by 2 investigators blind to treatments. Briefly, wakefulness was defined as desynchronized, low-amplitude EEG rhythms and high tonic EMG activity. NREM sleep was recognized as high-amplitude, low-frequency synchronized delta (1 to 4 Hz) waves and reduced EMG activity. REM sleep was defined as prominent consecutive theta (6 to 9 Hz) oscillations accompanied by further decreased EMG activity. The sleep/wakefulness states were manually corrected by an experimenter blind to treatments. REM sleep was carefully revised with 0.5-s increments to identify the REM sleep episode duration and the post-inter-REM sleep interval duration precisely. SOREM sleep-like behavior was defined by previous NREM sleep shorter than 60 s [27].

### Fiber photometry recordings and analyses

To observe the activity of the OX^SLD^ neurons/OX^PnO^ neurons/orexin-projecting ZI neurons, Ca^2+^ signals were recorded using a fiber photometry system (Inperstudio Alpha 8.2, Inper, China). During recordings, the optical fiber patch cord (diameter: 200 μm, NA: 0.37, Inper, China) with low auto-fluorescence was connected to an optical fiber. Two single-wavelength laser beams were used to excite GCaMP signals (488 nm) or Ca^2+^-independent fluorescent signals (405 nm, isosbestic wavelength), respectively. Both excitation wavelength powers were measured using a power meter (PM100D, Thorlabs, USA) and set to 30 to 40 μW at the end of the patch cord. The emitted signals were recorded at 30 Hz with alternating pulses of 488- and 405-nm light, resulting in 15 Hz sampling rate for the GCaMP and control signals. To synchronize fiber photometry recordings and EEG/EMG recordings, a Bayonet Neill–Concelman connector (BNC) cable carrying transistor-transistor-logic (TTL) pulses from the Inper system was connected to a digital input channel of the EEG/EMG recording system (Plexon, USA). Data from fiber photometry recordings were analyzed by MATLAB 2014a (MathWorks, USA). The sampled signals were low-pass filtered at 2 Hz with a zero-phase filter from the 2 excitation wavelengths, 488 and 405 nm. The filtered 405-nm signal was subtracted from the 488-nm signal using a least-squares linear fit. Then, the Δ*F*/*F* was calculated as (488-nm signal − fitted 405-nm signal)/(fitted 405-nm signal), and the obtained signals were *z*-scored for further analysis.

To observe Ca^2+^ fluorescence changes across state transitions, the time of state transitions between different vigilance states was first identified. Then, a total of 70-s GCaMP signals, including a 50-s pre-transition period and a 20-s post-transition period, were extracted and averaged for each tested mouse. A stable 10-s epoch (Δ*F*/*F*, *z*-scored) was chosen before and after state transitions for comparisons of the activity level among different states. We analyzed Ca^2+^ fluorescence changes for wake to NREM sleep, NREM sleep to wake, REM sleep to wake, and NREM to REM sleep transitions.

To analyze the activity dynamics of OX^SLD^ and OX^PnO^ neurons during REM sleep, in each tested mouse, GCaMP signals including 10-s NREM sleep and REM sleep episodes with different lengths (longer than 40 s, 60 s, and 100 s) were firstly extracted and averaged. Then, the GCaMP signals among individuals were further averaged to more clearly present the activity pattern during REM sleep. We found that GCaMP signals reached a steady activation level at ~30 s after entering REM sleep. Thus, to objectively measure the value of this activation, REM sleep episodes shorter than 30 s were first excluded. Further considering that a potential 5-s REM sleep–wakefulness transitional period may exist, we finally focused on those episodes longer than 45 s, which allowed an at least 10-s steady activation epoch for objective measurement. The orexin-projecting ZI neurons reached a steady activation at ~20 s, and thus, REM sleep episodes longer than 35 s were included. OX^SLD^ neurons during the rebound and recovery period after the RSD reached a steady activation at ~20 s and ~30 s, respectively, and REM sleep episodes longer than 35 s and 45 s were included. Then, the calculated value of the REM sleep steady activation in different neuronal populations was employed for correlation analyses with corresponding REM sleep core features, including EEG (theta/total and delta/total power) and EMG amplitudes. The correlation between this activation level and REM sleep episode duration was also analyzed. For analyzing the relationship between this activation level and the dissipation level of REM sleep pressure (Δ*P*) in the same REM sleep episode, we employed the post-inter-REM sleep interval. For a given REM sleep episode, Δ*P* = *P*_start_ − *P*_end_. *P*_start_ and *P*_end_ represent the REM sleep pressure level at REM sleep start or end respectively, and Δ*P* can be reflected directly by the changes in *P*_end_ because *P*_start_ is similar in stable conditions. *P*_end_ is thought to correlate with the duration of post-inter-REM sleep interval [71]. Actually, the post-inter-REM sleep interval duration directly reflects the time used to accumulate enough pressure for REM sleep triggering, and longer post-inter-REM sleep interval thus means a lower *P*_end_ in the preceding REM sleep episode. However, it should be noted that the accumulation during this time was reported to be affected by heterogeneous homeostatic processes, which should lead to a non-uniformity of the post-inter-REM sleep interval durations [71]. Therefore, the pressure level at the end of pre-REM sleep episodes (*P*_end_) cannot be uniformly reflected by all ranges of post-inter-REM sleep interval durations. We then only employed interval data shorter than 900 s, which were largely generated by a homogeneous type of regulation process, i.e., the short-term homeostatic process [72]. For a similar purpose, we also exclude abnormal intervals that are indicated by the following SOREM sleep-like behavior. Further considering that the trigger pressure for REM sleep start (*P*_start_) should be similar in stable conditions [9], the Δ*P* (*P*_start_ − *P*_end_) can be uniformly reflected by the post-inter-REM sleep interval durations within the range of 60 to 900 s and thus this range of data was used in the present study. In this condition, the contribution of steady activation level in different neuronal groups during REM sleep to Δ*P* can be meaningfully and objectively reflected by a correlation analysis.

### REM sleep deprivation

Mice were subjected to REM sleep deprivation (RSD) by employing the small platforms-over-water method [73]. Briefly, mice were placed on the 2 small-round platforms (diameter: 2.5 cm, 2 and 3 cm high) surrounded by a 1-cm-deep water in a Plexiglas barrel. RSD started at ZT 4 (12:00 AM). Mice had free access to food and water and kept under 12-h light/dark cycles. After 24-h RSD, mice were returned to their home cages, where they could sleep ad libitum for recovery. EEG/EMG recordings and fiber photometry recordings were conducted to monitor the sleep/wakefulness states and the Ca^2+^ signals, respectively.

### Sample of type I narcoleptic patients and analysis

We retrospectively reviewed all available type I narcoleptic patients diagnosed according to the International Classification of Sleep Disorders, third edition (ICSD-3) over a 4-year period (2019 to 2023) in the Sleep Medicine Center of West China Hospital. Clinical investigations were approved by West China Hospital Clinical Research Ethics Committee, and a written patient’s informed consent was obtained for all patients prior to participation. The included patients were diagnosed with type I narcolepsy at their first visit without drug treatment, and those with additional neurological or psychiatric disorders were excluded. To obtain clinical data during stable disease progression and largely reduce the influences of neuronal development, we included patients with age of narcolepsy onset older than 15 years and a disease duration larger than 1 year. A total of 41 Chinese patients (mean age: 36.1 ± 2.2 years; male: 73.2% [30/41]; female: 26.8% [11/41]) were included for analyses. We then extracted SOREM sleep data from their multiple sleep latency test (MLST). The number of SOREM sleep (latency ≤ 15 min) was calculated from a total of 5 standard MSLT trials during daytime naps, and the corresponding NREM to SOREM sleep latency was averaged for each patient. The score of cataplexy was calculated by the partial items from the UNS (i.e., from 0 to 24), and higher scores refer more frequent experiences of cataplectic attacks. For the obtained risk factors of narcolepsy comorbidity, the BMI was calculated as follows: body weight (kg)/height (m^2^). According to the Chinese BMI classification, obesity was defined with a BMI value ≥28 and overweight was defined with a value of 24 to 28. A BMI value lower than 24 was recognized as normal. The degree of depression and anxiety was measured by the SDS and Self-rating Anxiety Scale (SAS), respectively [74,75]. Briefly, the total raw scores of SDS or SAS ranged from 20 to 80, and the raw scores were converted into the depression or anxiety index (termed "SDS or SAS index"). The index score was used to judge the depression or anxiety degree, and a score ≥50 was diagnosed as depression or anxiety.

### Statistical analysis

All data were reported and plotted as mean ± SEM. Statistical analyses were performed in SigmaPlot 14.0 (Systat Software, USA) or Matlab 2014a (MathWorks, USA) if necessary. Briefly, a normality test was first examined on each dataset using the Shapiro–Wilk test. If the dataset passed the normality test, parametric tests (two-tailed paired *t* test, two-tailed unpaired *t* test, one-way repeated-measures analysis of variance [ANOVA], and one-way ANOVA) were used where appropriate. Otherwise, non-parametric tests (Wilcoxon signed-rank test, Mann–Whitney rank sum test, and Kruskal–Wallis one-way ANOVA on Ranks) were used where appropriate. *P* < 0.05 was accepted as statistically significant. The significance levels of data are denoted as *P < 0.05 and **P < 0.01. *P* > 0.05 was considered non-significant and was denoted as n.s.

## Data Availability

All data needed to evaluate the conclusions in the paper are present in the paper and/or the Supplementary Information. Additional data related to this paper may be requested from the authors.
