# Supplementary material for: Orexin Neurons to Sublaterodorsal Tegmental Nucleus Pathway Prevents Sleep Onset REM Sleep-Like Behavior by Relieving the REM Sleep Pressure
Source: Research (Wash D C). 2024 Apr 30;7:0355. doi: 10.34133/research.0355 (PMC11062508; doi:10.34133/research.0355)
Supplement: Supplementary 1 — Figs. S1 to S18 Tables S1 to S3 [file research.0355.f1.docx]

**Supplementary materials**

**Supplementary Figures and Figure Legends**

**Fig. S1. Location of the virus injections in the SLD.**

1. (Left panel) Representative coronal images from a retro-DIO-FLP/hSyn-EGFP (green) injected mouse showing that the virus diffusion is within the borders (bregma, AP: -4.96 to -5.34 mm) of the SLD region. All scale bars: 500 μm. (Right panel) Overlay for a series of viral injection regions from 4 mice.

Abbreviations: SLD, sublaterodorsal tegmental nucleus, scp, superior cerebellar peduncle; Mo5, motor trigeminal nucleus; 7n, facial nerve or its root.

**Fig. S2. Morphological validation of the projection-based viral-genetic labeling strategy.**

(A) Representative images showing the expression of mCherry^+^ (red) in orexin-A^+^ (blue) neurons in the LH. Scare bar, 100 μm.

(B) Quantification of the percentage of orexin-A^+^/mCherry^+^ neurons in all orexin-A^+^

neurons (SLD-projecting orexin neurons, 12.9 ± 1.5%) and the percentage of orexin-A^+^/mCherry^+^ neurons in all mCherry^+^ neurons (82.4 ± 0.8%) after the virus

infections (n = 3 mice). Abbreviation: f, fornix.

Data are presented as mean ± SEM.

**Fig. S3.** **The projection patterns of the orexin entirety in several important nodes.**

(A-F) Representative images showing the distribution of orexin-immunoreactive fibers in the SLD (A), VP (B), PVT (C), PnO (D), LC (E) and LDT (F). All scale bars, 100 μm. A magnified view of the indicated area (white box) is presented below. Scale bar, 20 μm.

(G) Group data showing the relative density of orexin-immunoreactive fibers in the VP, PVT, PnO, LC and LDT, compared with the SLD. (n = 4 mice).

Abbreviations: acp, anterior commissure, posterior; VP, ventral pallidum; 3V, 3rd ventricle; PVT, paraventricular thalamus nucleus; PnO, oral pontine reticular nucleus; Tz, nucleus of the trapezoid body; RPO, nucleus of the trapezoid body; LC, locus coeruleus; LDT, laterodorsal tegmental nucleus; Sph, sphenoid nucleus; DTgP, dorsal tegmental nucleus, pericentral part.

Data are presented as mean ± SEM.

**
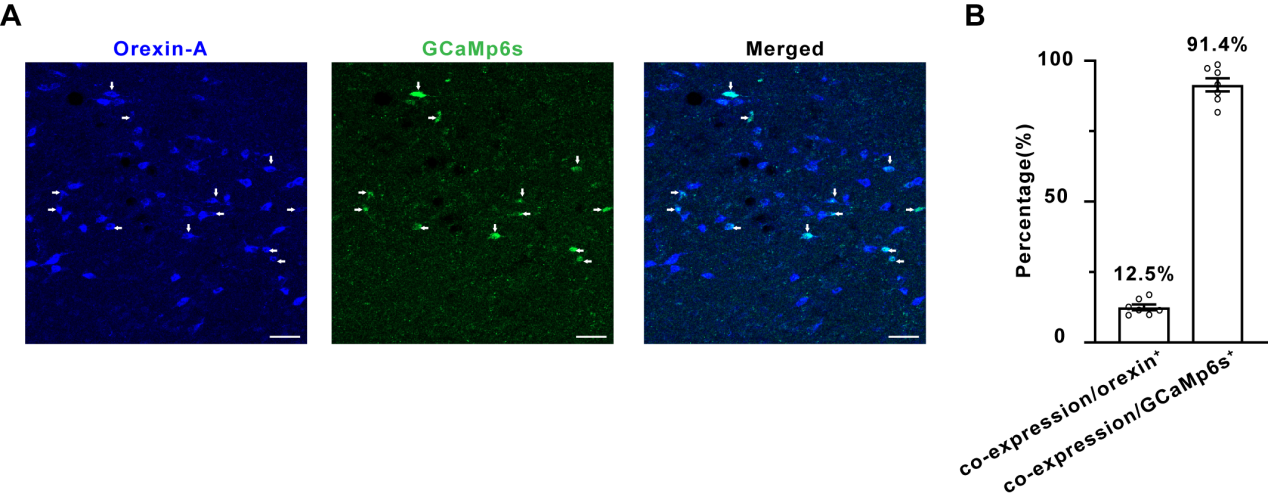
**

**Fig. S4. Morphological validation of fiber photometry recordings.**

(A) Representative images showing the expression of GCaMp6s^+^ (green) in orexin-A^+^ (blue) neurons in the LH. Scare bar, 50 μm.

(B) Quantification of the percentage of orexin-A^+^/GCaMp6s^+^ neurons in all orexin-A^+^

neurons (SLD-projecting orexin neurons, 12.5 ± 1.0%) and the percentage of

orexin-A^+^/GCaMp6s^+^ neurons in all mCherry^+^ neurons (91.4 ± 2.4%) after the virus

infections (n = 7 mice).

Data are presented as mean ± SEM.


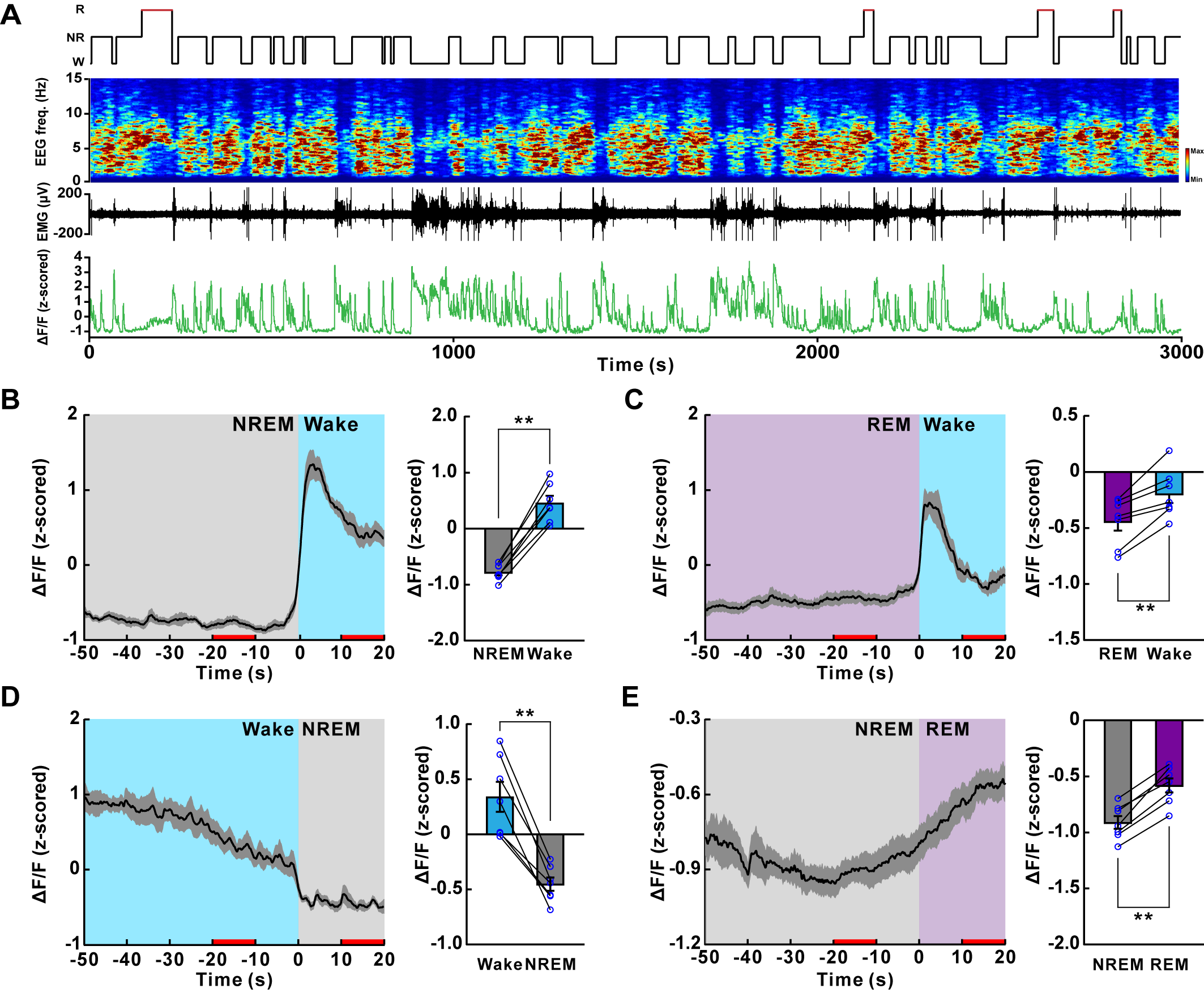


**Fig. S5.** **The activity dynamics of OX^SLD^ neurons during sleep/wakefulness transitions.**

(A) Representative hypnogram, EEG spectrograms, EMG trace, and GCaMP fluorescence trace of OX^SLD^ neurons recorded simultaneously over time across different sleep/wakefulness states.

(B-E) Averaged OX^SLD^ neuronal activity across NREM sleep to wakefulness transitions (B), REM sleep to wakefulness transitions (C), wakefulness to NREM sleep transitions (D) and NREM to REM sleep transitions (E). Left panels: averaged traces; Right panels: comparisons between the activity level before and after transitions. All activity traces are z-scored. Red lines indicate the time periods chosen for comparison. Shadow represents SEM (n = 7 mice).

Abbreviations: PnC, caudal pontine reticular nucleus; DMTg, dorsomedial tegmental area.

Data are presented as mean ± SEM. **P < 0.01, two-tailed paired t-test (B-E).

**
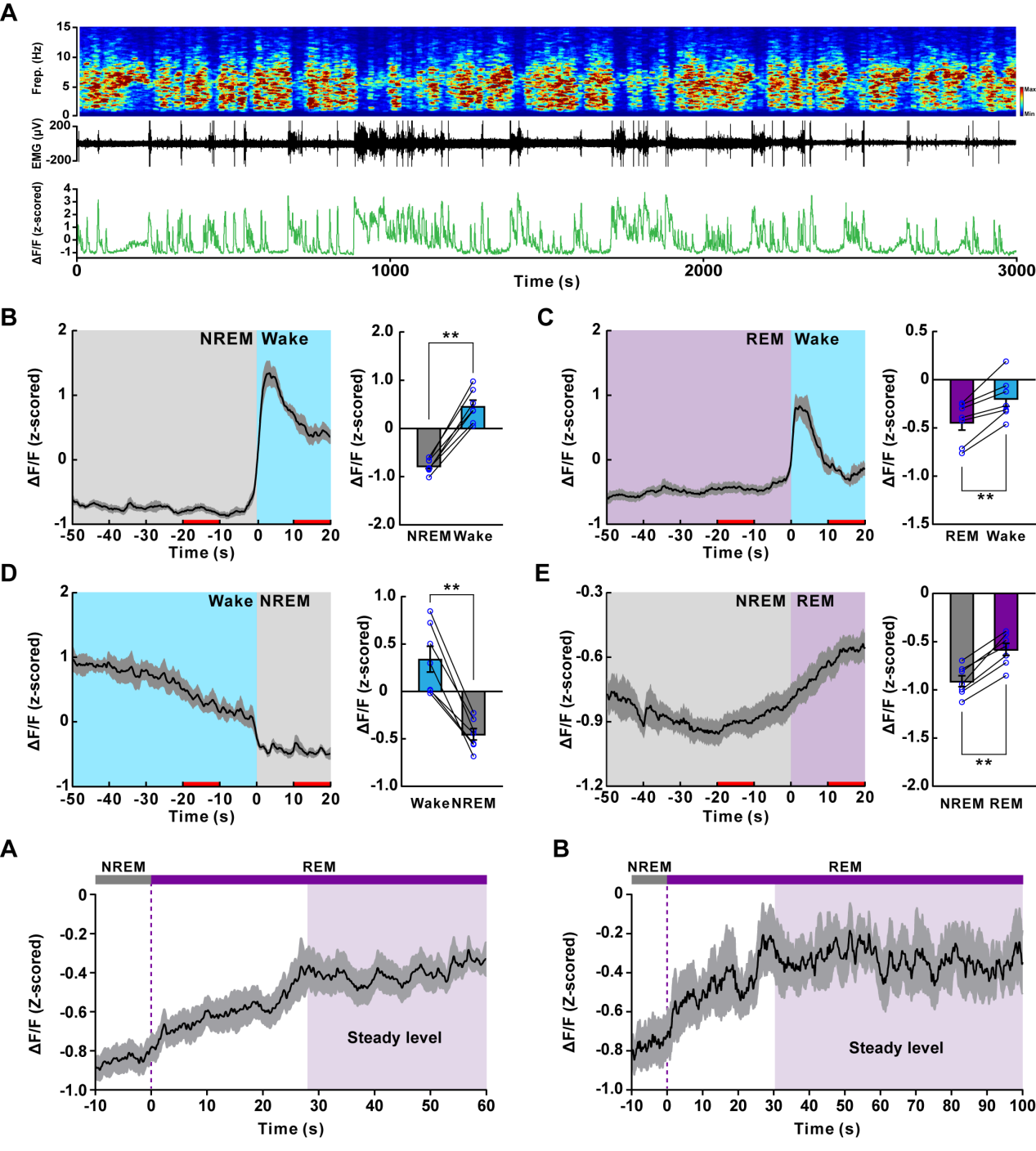
**

**Fig. S6. The activity pattern of OX^SLD^ neurons during REM sleep episodes.**

(A and B) Averaged OX^SLD^ neuronal activity (ΔF/F, z-scored) during the REM sleep episodes with a duration longer than 60 s (A, n = 7 mice) or 100 s (B, n = 5 mice) and the preceding 10-s NREM sleep episodes. Shadow represents SEM.

**
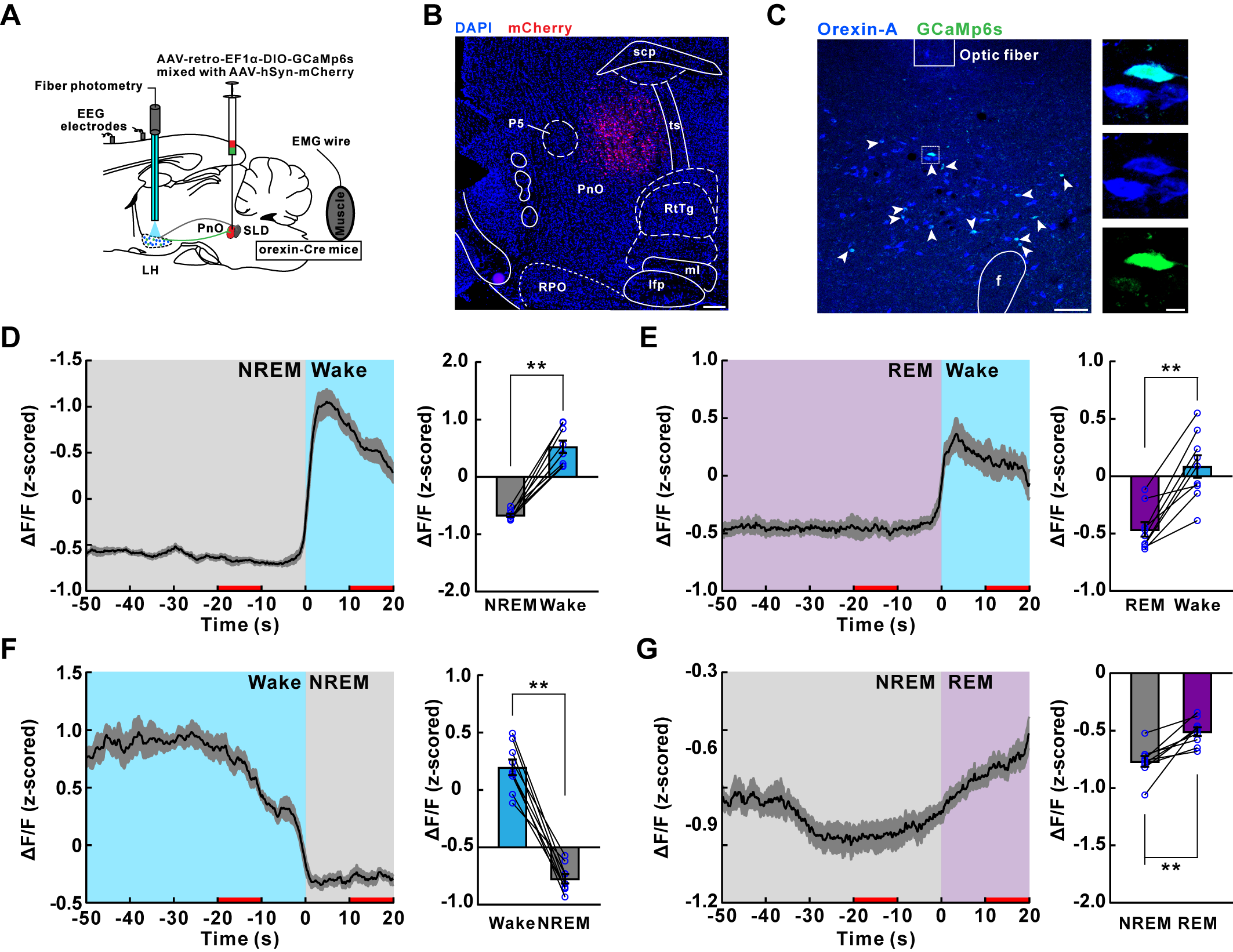
**

**Fig. S7. The activity dynamics of OX^PnO^ neurons during sleep/wakefulness transitions.**

1. Schematic drawing of fiber photometry recordings in the LH OX^PnO^ neurons.
2. A representative image showing virus injection sites in the PnO. Scale bar, 200 μm.
3. Representative images showing GCaMp6s^+^ expression (green) in orexin-A^+^ neurons (blue). Scale bars, 100 μm (merged view) or 10 μm (magnified view).
4. G) Averaged OX^PnO^ neuronal activity across NREM sleep to wakefulness transitions (D), REM sleep to wakefulness transitions (E), wakefulness to NREM sleep transitions (F), and NREM to REM sleep transitions (G). Left panel: averaged traces; Right panel: comparisons between the activity level before and after transitions. All activity traces are z-scored. Red lines indicate the time periods chosen for comparison. Shadow represents SEM (n = 9 mice).

Abbreviations: P5, peritrigeminal zone; scp, superior cerebellar peduncle; ts, tectospinal tract ; RtTg, reticulotegmental nucleus of the pons; ml, medial lemniscus; lfp, longitudinal fasciculus of the pons; RPO, rostral periolivary region.

Data are presented as mean ± SEM. **P < 0.01, two-tailed paired t-test (D-G).

**
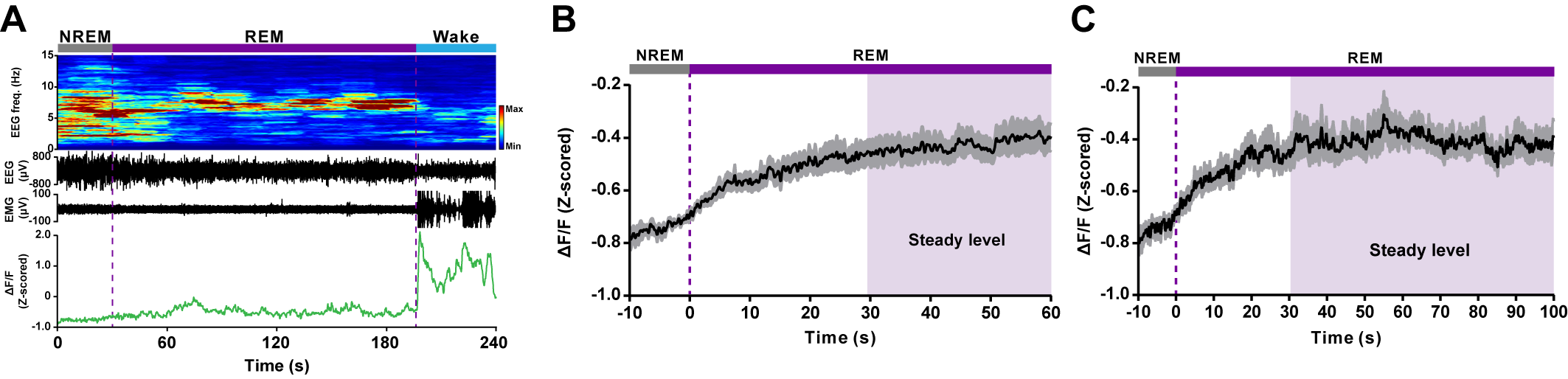
**

**Fig. S8.** **The activity pattern of** **OX^PnO^ neurons during REM sleep episodes.**

(A) A representative color-coded hypnogram, EEG spectrograms, EEG/EMG traces, and GCaMP fluorescence trace (ΔF/F, z-scored) of OX^PnO^ neurons recorded simultaneously in an episode of REM sleep with the preceding NREM sleep and following wakefulness.

(B and C) The averaged OX^PnO^ neuronal activity (ΔF/F, z-scored) during REM sleep episodes with a duration longer than 60 s (B, n = 9 mice) or 100 s (C, n = 9 mice) and the preceding 10-s NREM sleep episodes. Shadow represents SEM.


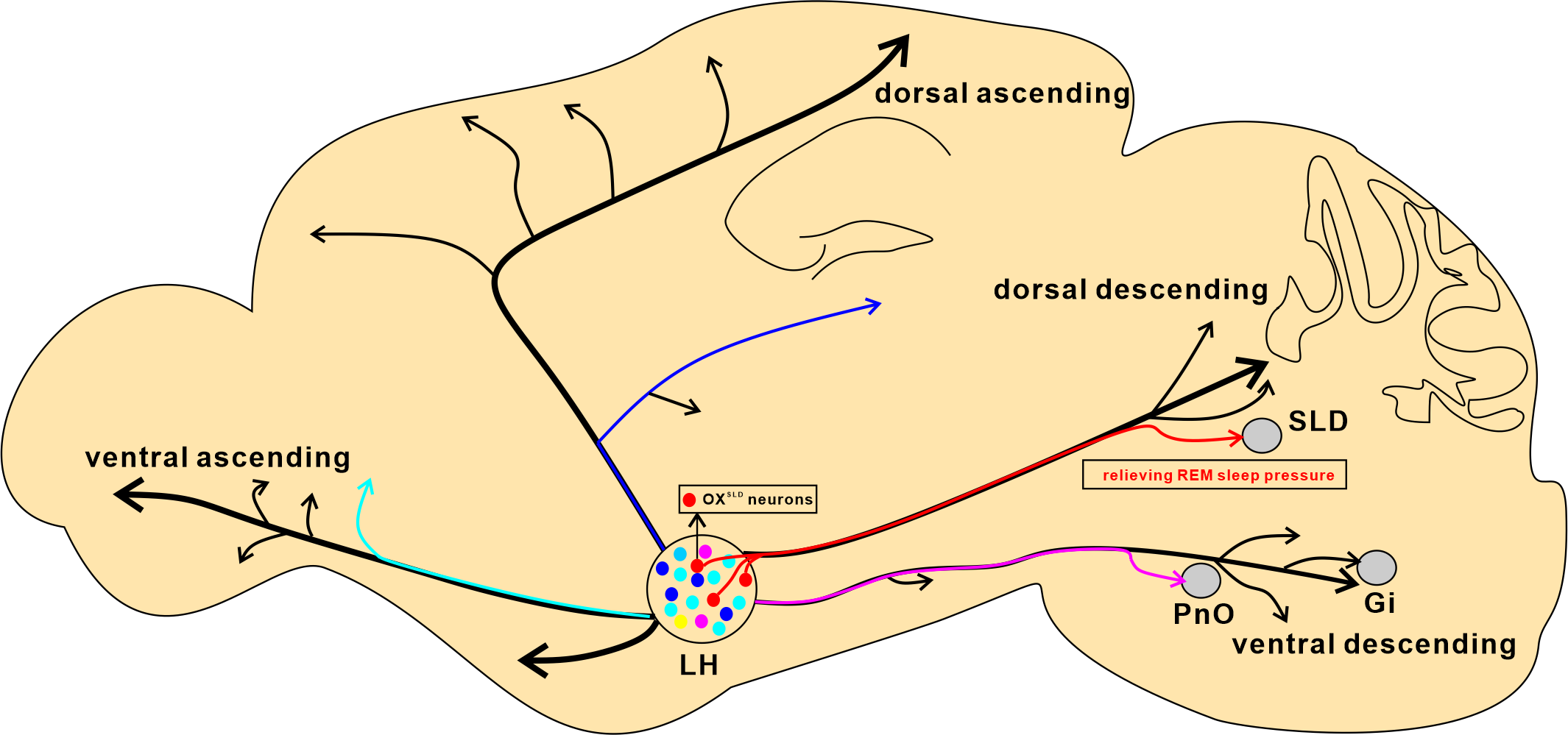


**Figure S9. A schematic diagram illustrating the organization of distinct orexin subgroups based on their projections to function-related regions, with orexin neurons projecting to the SLD playing a specific role in relieving REM sleep pressure.**

This schematic presents an organization notion for different functional subgroups of LH orexin neurons (indicated by different colored circles), which might be organized according to their distinct projections to different functional brain areas. This kind of notion has been proposed in previous studies that focus on the roles of orexin neurons in reward-seeking, wakefulness regulation, and motor control (Harris et al., Nature, 2005; Hu et al., Neurosci Biobehav Rev. 2015; Iyer et al., J Comp Neurol. 2018). Importantly, for REM sleep regulation, our series of works have found several subgroups of orexin neurons, including the OX^SLD^, OX^PnO^, and OX^Gi^ neurons. Intriguingly, only the OX^SLD^ is specifically involved in relieving REM sleep pressure.


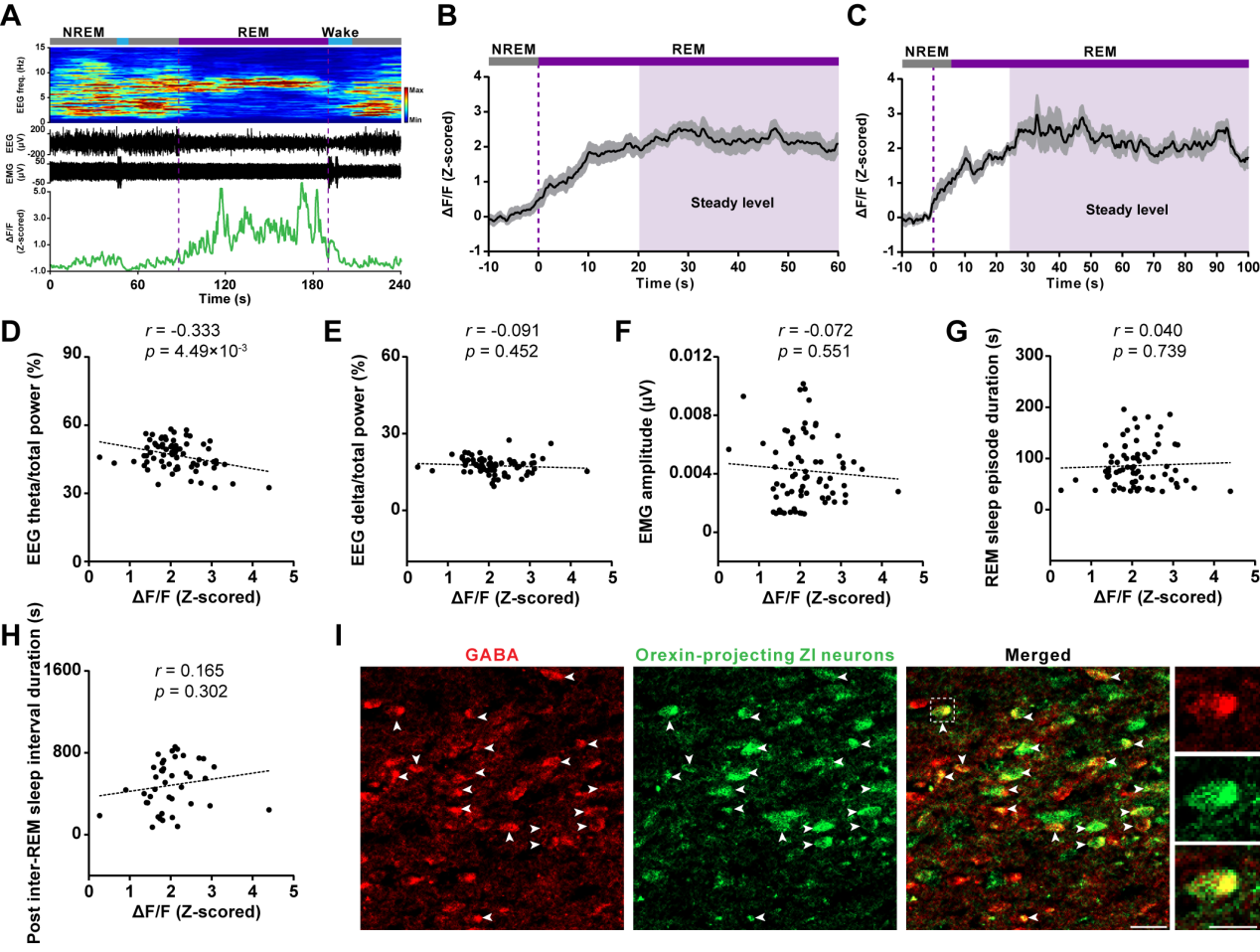


**Fig. S10. Activity pattern of orexin-projecting ZI neurons during REM sleep and their correlation with REM sleep regulation.**

(A) Representative color-coded hypnogram, EEG spectrograms, EEG/EMG traces and GCaMP fluorescence trace of orexin-projecting ZI neurons recorded simultaneously in a REM sleep episode.

(B and C) The averaged orexin-projecting ZI neuronal activity (ΔF/F, z-scored) during the REM sleep episodes with a duration longer than 60 s (B, n = 6 mice) or 100 s (C, n = 6 mice) and the preceding 10-s NREM sleep episodes. Shadow represents SEM.

1. G) Correlation between the steady activation level of orexin-projecting ZI neurons during REM sleep episodes and the corresponding EEG theta/total power (D), delta/total power (E), integrated EMG amplitude (F), REM sleep episode duration (G) (n = 71 episodes from 6 mice). Dashed line, linear fit.
2. Correlation between the steady activation level of orexin-projecting ZI neurons during REM sleep episodes and the uniformly generated post inter-REM sleep interval duration (n = 41 episodes from 6 mice). Dashed line, linear fit.
3. Representative images showing that the majority of orexin-projecting ZI neurons (green) were co-localized with GABA immunoreactivity (red). Scale bars, 20 μm (low-magnified view) or 10 μm (magnified view).

**
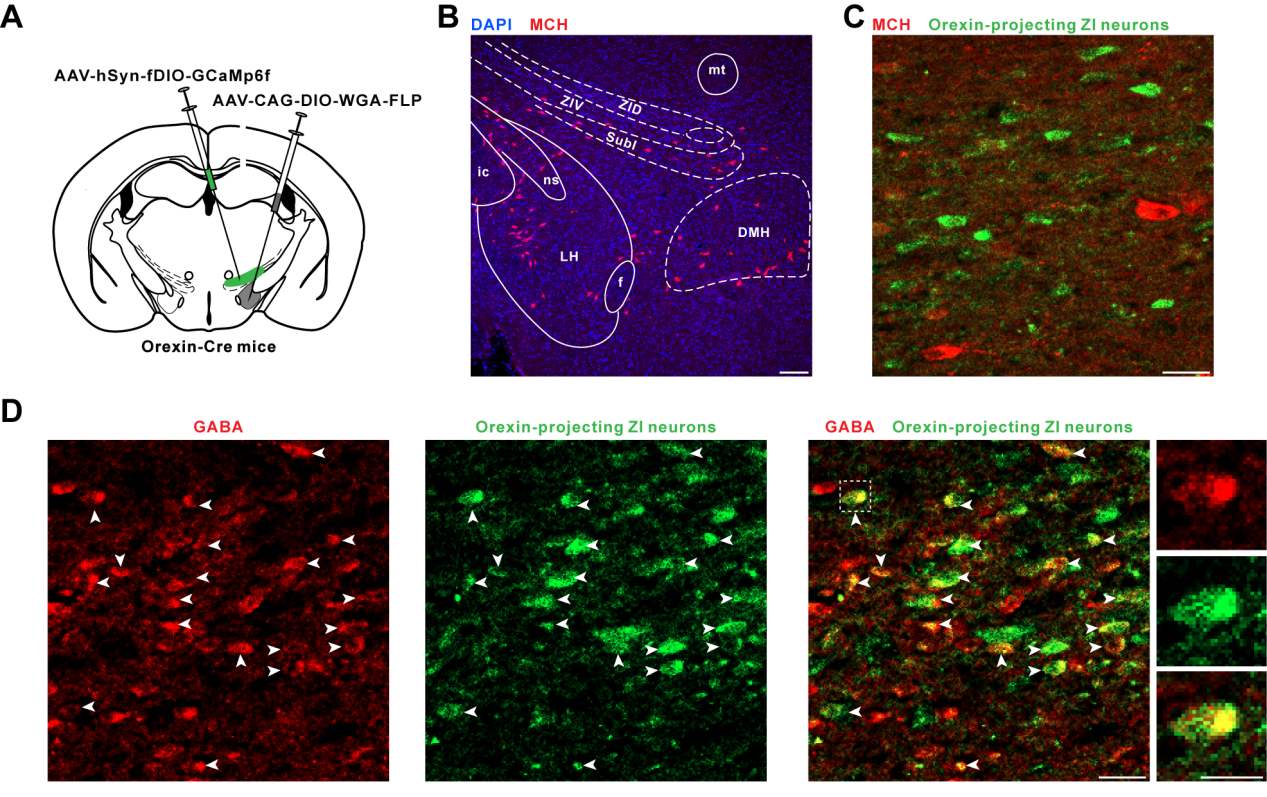
**

**Fig. S11. Orexin-projecting ZI neurons were not co-localized with MCH immunoreactivity.**

1. Schematic of virus injections showing retrograde tracing of orexin-projecting ZI neurons.
2. A representative image showing MCH-positive cell bodies were exclusively located in the LH, DMH and ZI nuclei. Scale bar, 100 μm.
3. A representative image showing orexin-projecting ZI neurons (green) were not co-localized with MCH immunoreactivity (red). Scale bar, 20 μm.

**
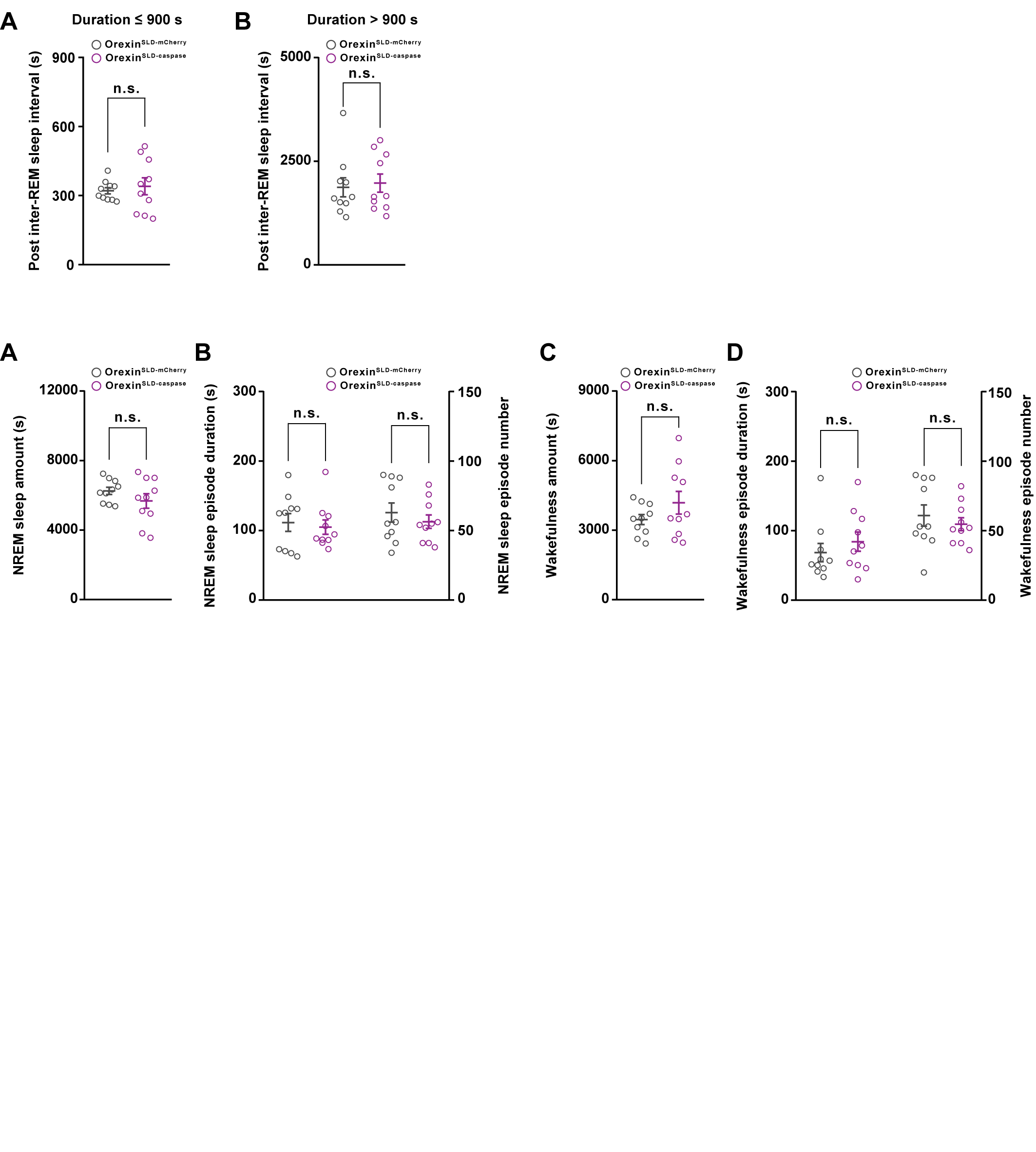
**

**Fig. S12.** **Influences of orexin-SLD pathway ablation on the post inter-REM sleep interval duration.**

(A and B) Changes of the post inter-REM sleep interval duration less than or equal to 900 s (A) and longer than 900 s (B) during the 3-h recording period in orexin^SLD-mCherry^ mice and orexin^SLD-caspase^ mice (n = 10 mice for each group).

Data are presented as mean ± SEM.

**
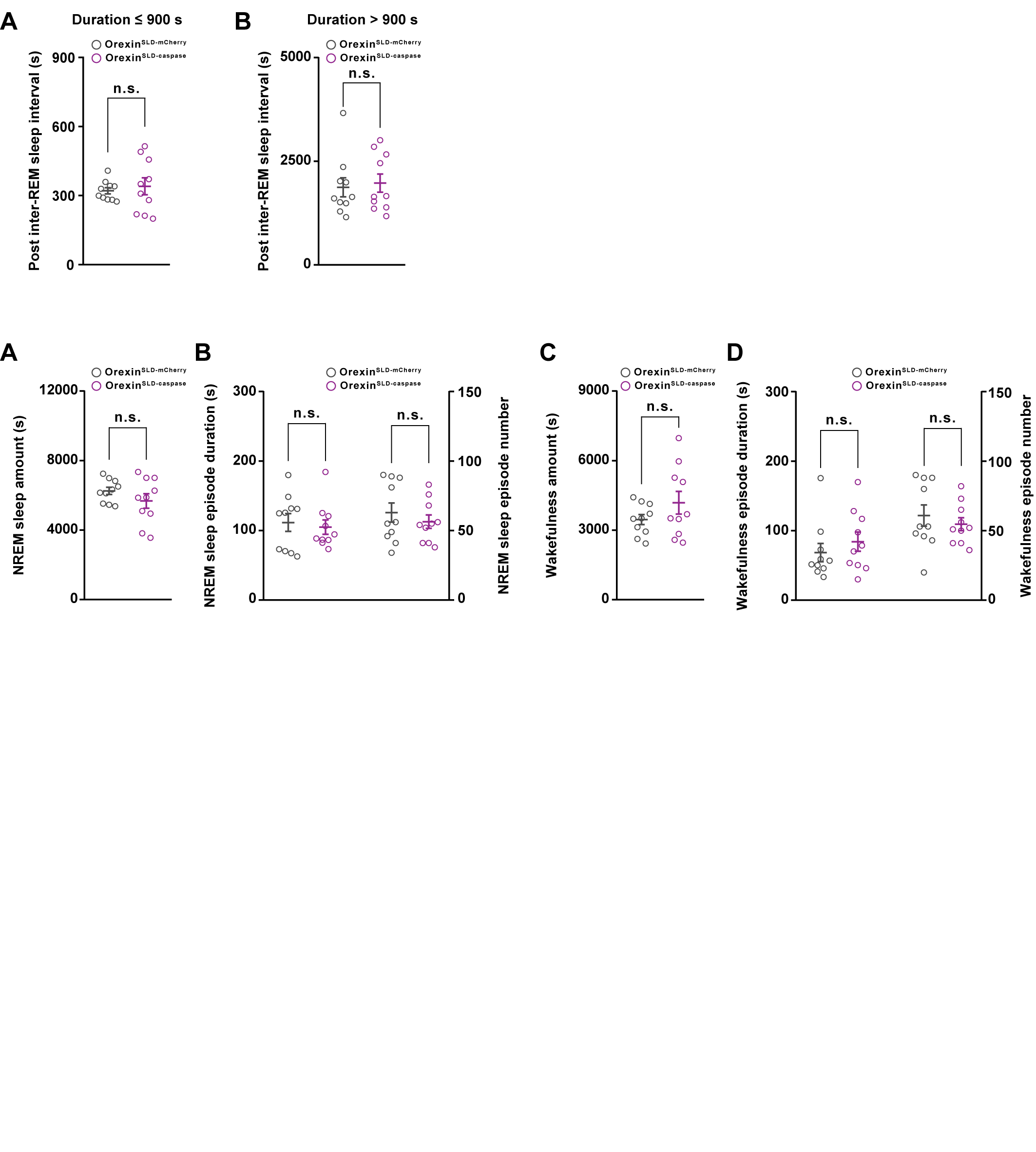
**

**Fig. S13.** **Influences of orexin-SLD pathway ablation on the NREM sleep and wakefulness states.**

(A and B) Changes of the NREM sleep amount (A), NREM sleep episode duration (B, left) and number (B, right) during the 3-h recording period between orexin^SLD-mCherry^ mice and orexin^SLD-caspase^ mice (n = 10 mice for each group).

(C and D) Changes of the wakefulness amount (C), wakefulness episode duration (D, left) and number (D, right) during the 3-h recording period between orexin^SLD-mCherry^ mice and orexin^SLD-caspase^ mice (n = 10 mice for each group).

Data are presented as mean ± SEM.

**
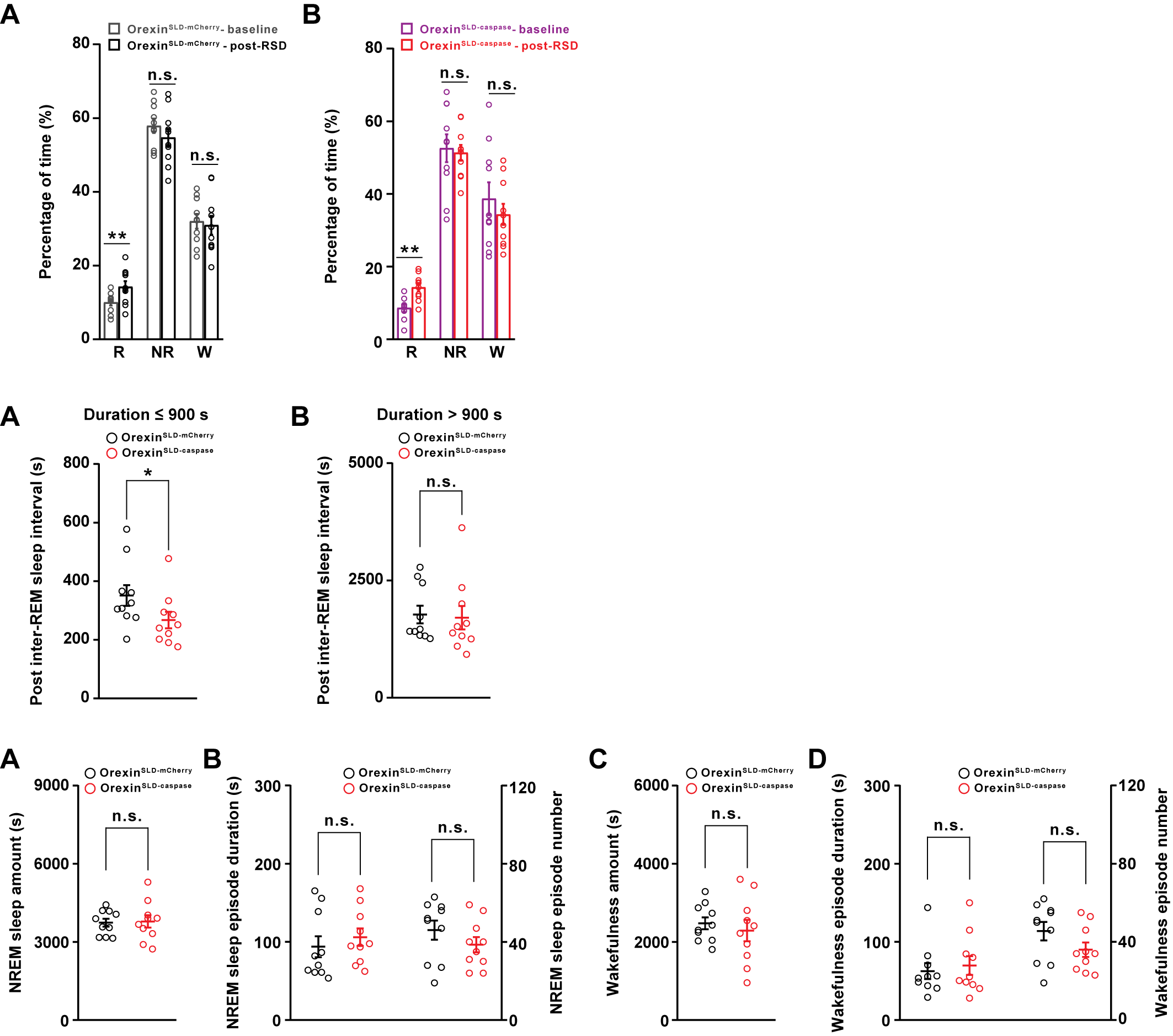
**

**Fig. S14. Influences of 24-h REM sleep deprivation on the vigilance states.**

(A) Percentage of time spent in REM sleep, NREM sleep and wakefulness during the 3-h recording period in the baseline and the recovery condition after 24h RSD of orexin^SLD-mCherry^ mice (n = 10 mice for each group).

(B) Percentage of time spent in REM sleep, NREM sleep and wakefulness during the 3-h recording period in the baseline and the recovery condition after 24h RSD of orexin^SLD-caspase^ mice (n = 10 mice for each group).

Data are presented as mean ± SEM. **P < 0.01, two-tailed paired t-test (A and B).


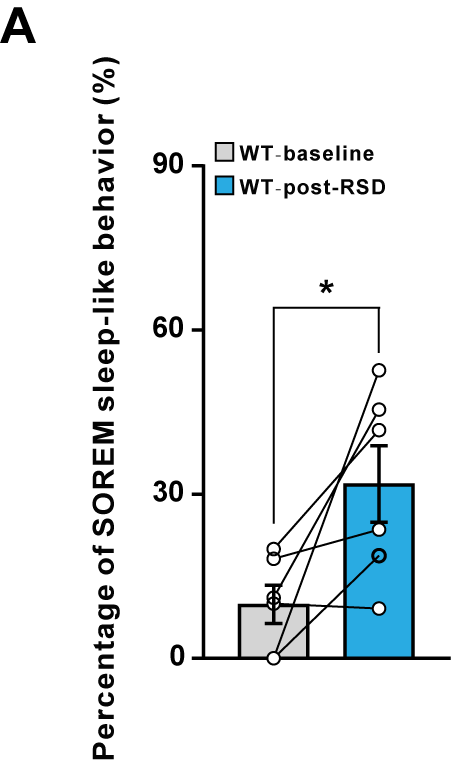


**Fig. S15. Influences of 24-h REM sleep deprivation on the percentage of SOREM sleep-like behavior of wild-type mice.**

1. The percentage of SOREM sleep-like behavior was significantly increased after 24-RSD (n = 6 mice).

Data are presented as mean ± SEM. *P < 0.05, two-tailed paired t-test.

**
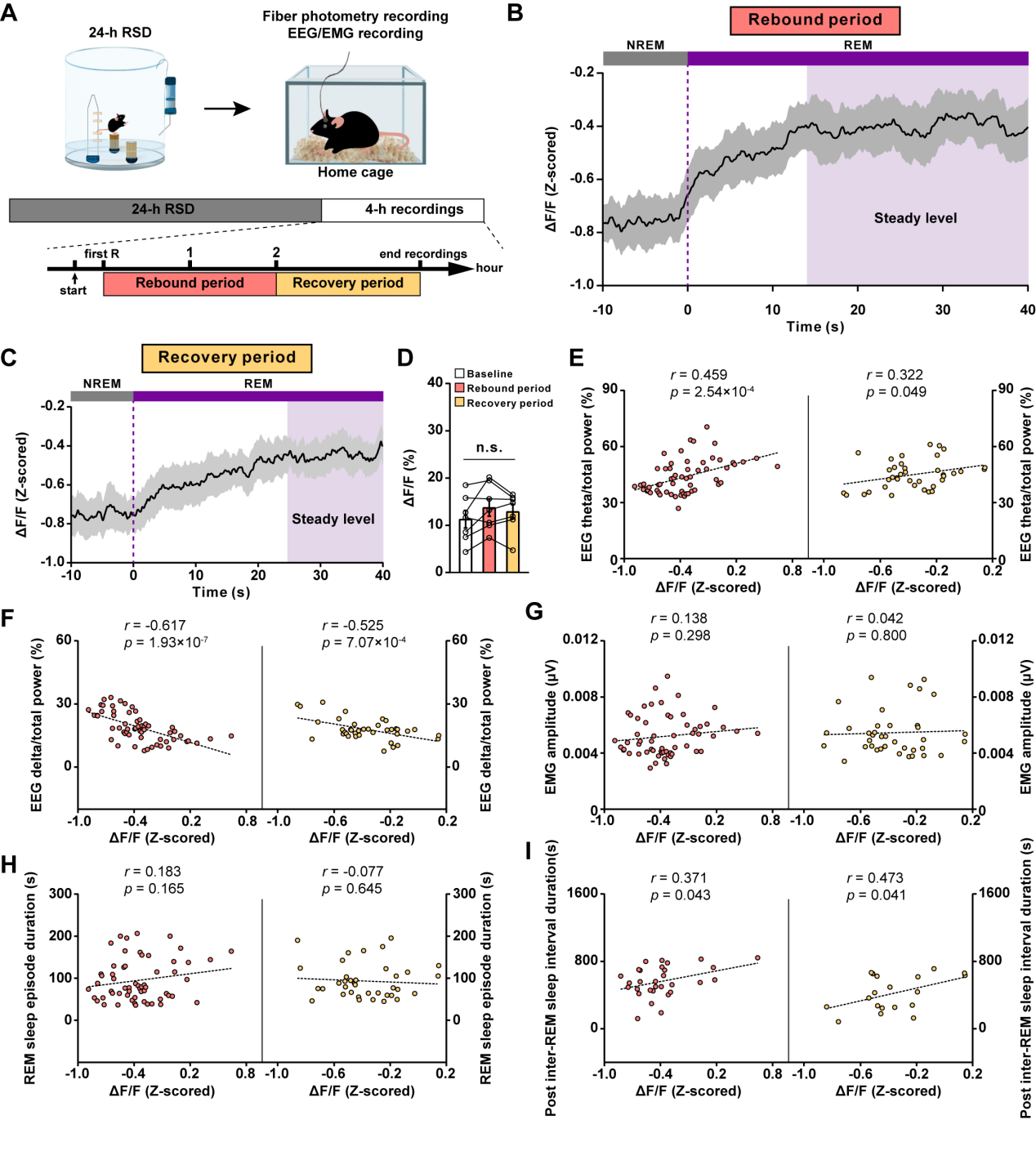
**

**Fig. S16. The activity of OX^SLD^ neurons during the REM sleep rebound period and the following recovery period and their REM sleep related functions.**

(A) Schematic drawing of 24-h REM sleep deprivation (RSD) using the small-platforms-over-water method followed by fiber photometry recordings and EEG/EMG recordings in the home cage (upper panel). Bottom panel showing procedures for 4-h recordings. The time period of 2 hours following the first REM sleep was defined as the rebound period. The remaining time until recordings ended was defined as the recovery period.

(B) Averaged OX^SLD^ neuronal activity (ΔF/F, z-scored) during the rebound period, and note that OX^SLD^ neurons reached the steady activation level earlier (~ 20 s) after entering REM sleep in this high-need condition (n = 7 mice). Shadow represents SEM.

(C) Averaged OX^SLD^ neuronal activity (ΔF/F, z-scored) during the recovery period, and note that the time (~ 30 s) to reach the steady activation level recovered in this period (n = 7 mice). Shadow represents SEM.

(D) Statistics of the averaged steady activation level (ΔF/F) in OX^SLD^ neurons during REM sleep episodes in baseline, rebound and recovery period, respectively (n = 7 mice, F (2, 12) = 2.821, P = 0.099).

(E) The significant positive correlation between the steady activation level of OX^SLD^ neurons with the corresponding EEG theta/total power during REM sleep still existed in the rebound (n = 59 episodes from 7 mice) and recovery period (n = 38 episodes from 7 mice).

(F) The significant negative correlation between the steady activation level of OX^SLD^ neurons with the corresponding EEG delta/total power during REM sleep still existed in the rebound (n = 59 episodes from 7 mice) and recovery period (n = 38 episodes from 7 mice).

(G) No significant correlation between the steady activation level of OX^SLD^ neurons with the corresponding integrated EMG amplitude during REM sleep was found in the rebound (n = 59 episodes from 7 mice) and recovery period (n = 38 episodes from 7 mice).

(H) No significant correlation between the steady activation level of OX^SLD^ neurons with the REM sleep episode duration was found in the rebound (n = 59 episodes from 7 mice) and recovery period (n = 38 episodes from 7 mice).

(I) Similar to normal conditions, there was an obvious positive correlation between the steady activation level of OX^SLD^ neurons and the post inter-REM sleep interval duration in the rebound (n = 30 episodes from 7 mice) and recovery period (n = 19 episodes from 7 mice). These results suggest that OX^SLD^ neurons could also actively contribute to relieving REM sleep pressure in this high-need condition.

Data are presented as mean ± SEM.

**
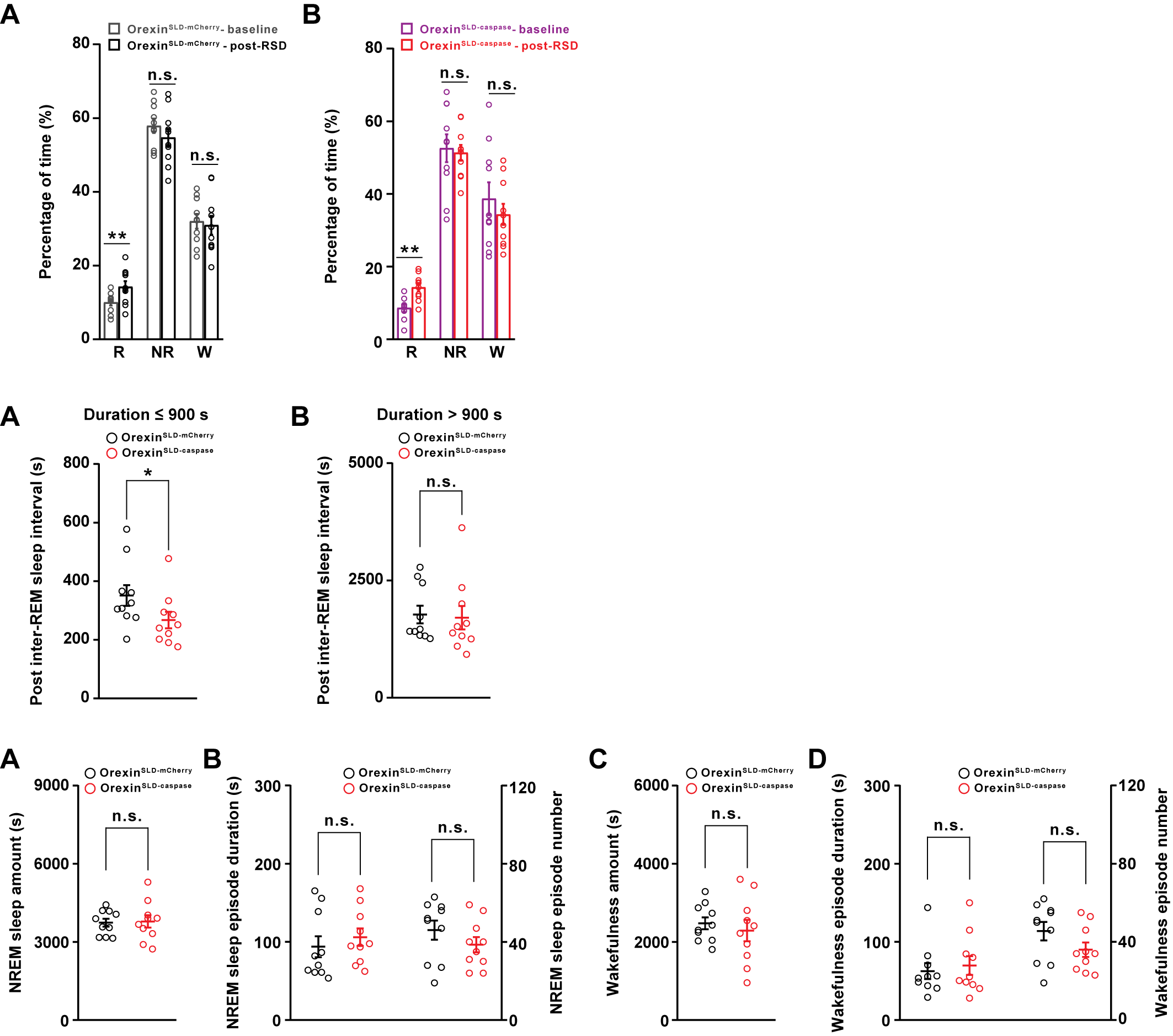
**

**Fig. S17. Influences of the orexin-SLD pathway ablation on the post inter-REM sleep interval duration in REM sleep high-need condition.**

(A and B) Changes of the post inter-REM sleep interval duration less than or equal to 900 s (A) and longer than 900 s (B) during 2-h rebound period after 24-h REM sleep deprivation in orexin^SLD-mCherry^ mice and orexin^SLD-caspase^ mice (n = 10 mice for each group).

Data are presented as mean ± SEM. *p < 0.05, two-tailed Mann-Whitney rank sum test (A).

**
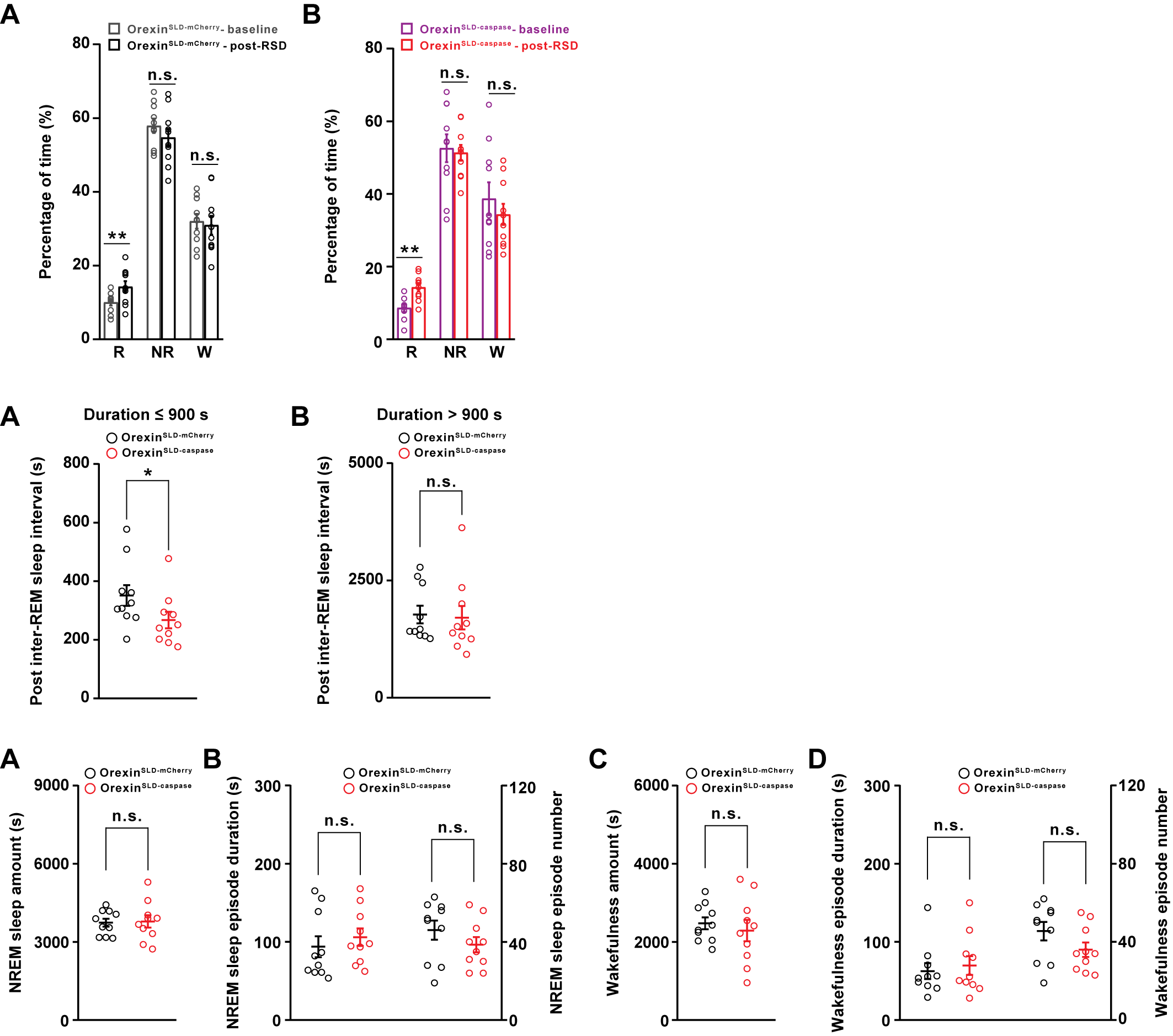
**

**Fig. S18. Influences of the orexin-SLD pathway ablation on the NREM sleep and wakefulness states in REM sleep high-need condition.**

(A and B) Changes of the NREM sleep amount (A), NREM sleep episode duration (B, left) and number (B, right) during 2-h rebound period after 24-h REM sleep deprivation between orexin^SLD-mCherry^ mice and orexin^SLD-caspase^ mice (n = 10 mice for each group).

(C and D) Changes of the wakefulness amount (C), wakefulness episode duration (D, left) and number (D, right) during 2-h rebound period after 24-h REM sleep deprivation between orexin^SLD-mCherry^ mice and orexin^SLD-caspase^ mice (n = 10 mice for each group).

Data are presented as mean ± SEM.
